# The MAP kinase MpkA controls cell wall integrity, oxidative stress response, gliotoxin production and iron adaptation in *Aspergillus fumigatus*

**DOI:** 10.1111/j.1365-2958.2011.07778.x

**Published:** 2011-08-30

**Authors:** Radhika Jain, Vito Valiante, Nicole Remme, Teresa Docimo, Thorsten Heinekamp, Christian Hertweck, Jonathan Gershenzon, Hubertus Haas, Axel A Brakhage

**Affiliations:** 1 Department of Molecular and Applied MicrobiologyBeutenbergstrasse 11a, D-07745 Jena, Germany; 2 Department of Biomolecular Chemistry, Leibniz Institute for Natural Product Research and Infection Biology (HKI)Beutenbergstrasse 11a, D-07745 Jena, Germany; 3 Friedrich Schiller University JenaBeutenbergstrasse 11a, D-07745 Jena, Germany; 4 Department of Biochemistry, Max Planck Institute for Chemical EcologyHans Knoell Str. 8, D-07745 Jena, Germany; 5 Division of Molecular Biology/Biocenter, Medical University InnsbruckFritz-Pregl-Str. 3, A-6020 Innsbruck, Austria

## Abstract

The saprophytic fungus *Aspergillus fumigatus* is the most important air-borne fungal pathogen. The cell wall of *A. fumigatus* has been studied intensively as a potential target for development of effective antifungal agents. A major role in maintaining cell wall integrity is played by the mitogen-activated protein kinase (MAPK) MpkA. To gain a comprehensive insight into this central signal transduction pathway, we performed a transcriptome analysis of the Δ*mpkA* mutant under standard and cell wall stress conditions. Besides genes involved in cell wall remodelling, protection against ROS and secondary metabolism such as gliotoxin, pyomelanin and pseurotin A, also genes involved in siderophore biosynthesis were regulated by MpkA. Consistently, northern and western blot analyses indicated that iron starvation triggers phosphorylation and thus activation of MpkA. Furthermore, localization studies indicated that MpkA accumulates in the nucleus under iron depletion. Hence, we report the first connection between a MAPK pathway and siderophore biosynthesis. The measurement of amino acid pools and of the pools of polyamines indicated that arginine was continuously converted into ornithine to fuel the siderophore pool in the Δ*mpkA* mutant strain. Based on our data, we propose that MpkA fine-tunes the balance between stress response and energy consuming cellular processes.

## Introduction

Responding to external signals and adaptation to changes in the environment is indispensable for the viability of all organisms. *Aspergillus fumigatus*, a saprophytic fungus, is able to grow and proliferate in a variety of environments (Tekaia and Latgé, 2005). It can colonize the human body where it is challenged by diverse physical and chemical conditions, such as presence of cell wall-degrading enzymes, oxidizing agents, limited nutrient availability, or changing pH, osmolarity and temperature. Yet, it has emerged to be the most common fungal pathogen causing invasive aspergillosis (IA) in immunocompromised patients (Brakhage, 2005; Dagenais and Keller, 2009; Brakhage *et al*., 2010).

Fungal pathogens such as *A. fumigatus* employ signal transduction cascades including mitogen-activated protein kinase (MAPK) pathways to sense, transduce and regulate different developmental processes of the fungal cell in response to extracellular cues (Rispail *et al*., 2009). The genome of *A. fumigatus* harbours four MAPK genes: *sakA/hogA*, *mpkA*, *mpkB* and *mpkC* (May *et al*., 2005). MpkA is a key player of the cell wall integrity pathway of *A. fumigatus*. In addition, it is involved in response to reactive oxygen species (ROS) and affects the formation of pyomelanin derived from tyrosine degradation (Valiante *et al*., 2008; 2009). The MAP kinase SakA has been shown to be involved in conidial germination under nitrogen and carbon source starvation and to respond to hypertonic conditions, ROS and heat shock (Xue *et al*., 2004). MpkC shows similarity to SakA. This kinase is involved in sensing alternative carbon sources (Reyes *et al*., 2006). Notably, MpkB has not been characterized yet.

The canonical MAPK pathway contains a central, three-tiered module comprising a MAP kinase kinase kinase (MAPKKK), a MAP kinase kinase (MAPKK) and the final MAP kinase, which is activated by dual phosphorylation of conserved threonine and tyrosine residues (Chang and Karin, 2001). In this activation loop, the cell wall plays an important role both as sensor and as protector of the living cell. It is subjected to continuous changes in its polymer composition in response to different environmental stimuli. Furthermore, the composition of the fungal cell wall is unique to eukaryotic organisms and therefore is considered an attractive target in antifungal drug research (Gastebois *et al*., 2009). Thus, knowledge about the role of signal transduction pathways for the rigidity of the cell wall is of great importance.

Despite the significance of the cell wall as an antifungal target, comparatively few classes of cell wall inhibitors have been used in a clinical set up (Walker *et al*., 2010). It was observed that clinical isolates of *Aspergillus* spp. are resistant against potent cell wall-disturbing agents like echinocandins (Arendrup *et al*., 2008). While these compounds are effective against *A. fumigatus in vitro*, information is still lacking concerning the pharmacodynamic for echinocandin monotherapy in patients with IA (Lewis, 2009). It is conceivable that salvage pathways exist in *A. fumigatus* that could reduce the efficacy of fungal drugs targeted against the cell wall. On the other hand, recent studies on a mutant lacking *mkk2* (MAPKK) of the cell wall integrity pathway in *A. fumigatus* showed an enhanced sensitivity to azoles, namely posaconazole and voriconazole (Dirr *et al*., 2010). In view of these facts, it becomes crucial to understand the wide-ranging role played by the cell wall integrity pathway in fungal pathogens such as *A. fumigatus*.

Here, we report a comprehensive study about the role of the cell wall integrity pathway in *A. fumigatus*. We found that MpkA is involved not only in co-ordinating cell wall remodelling, but also plays an important role in the response against ROS and for secondary metabolite production. Even more, MpkA is specifically involved in siderophore production during iron starvation. This is the first report linking a MAP kinase pathway with siderophore production. Taken together, our data indicate that MpkA represents a global regulator in *A. fumigatus*.

## Results

### Transcriptome analysis

**Fig. 1:**
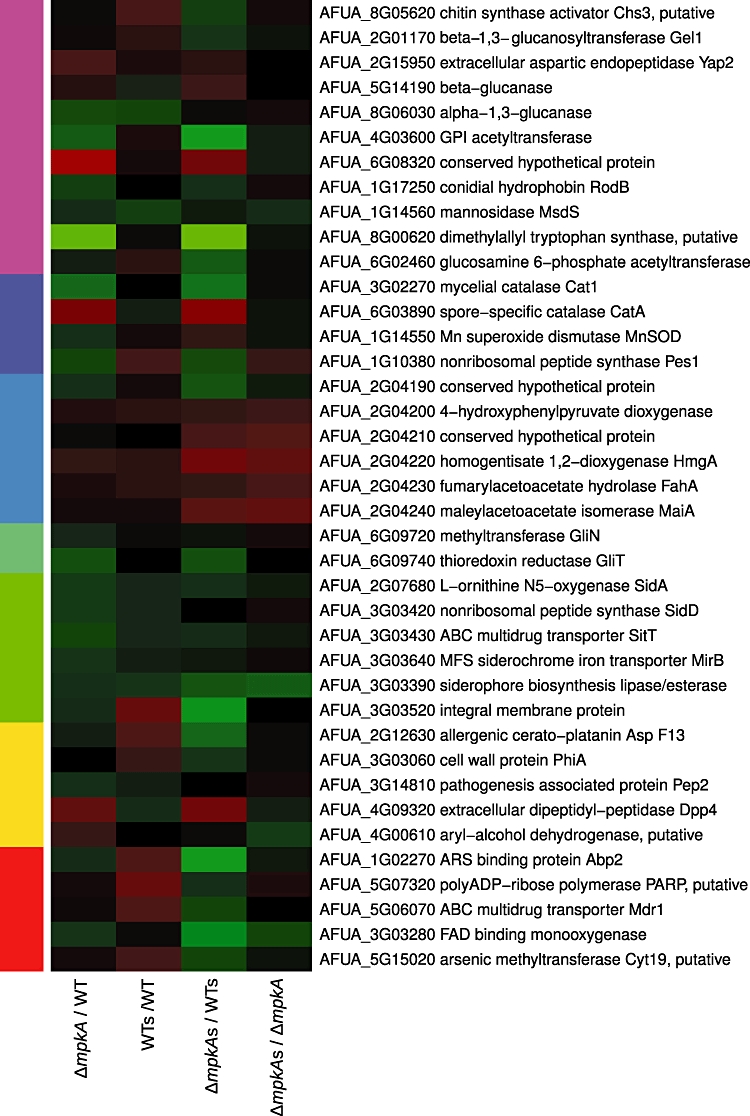
Heatmap showing the pattern of expression of *A. fumigatus* selected genes in four comparisons. Column 1, Δ*mpkA/*wt (time point 0 min); column 2, wt + glucanex (time point 20 min)/wt; column 3, Δ*mpkA* + glucanex (time point 20 min)/wt + glucanex (time point 20 min); column 4, Δ*mpkA* + glucanex (time point 20 min)/Δ*mpkA* (time point 0 min). The average expression ratios on a Log_2_ scale are colour coded as indicated by the bar above. Abbreviations: Δ*mpkA/*wt – fold change between Δ*mpkA* and wild type; wt + glucanex/wt – fold change between wild type under glucanex stress and wild type without stress; Δ*mpkA* + glucanex/wt + glucanex – fold change between Δ*mpkA* under glucanex stress and wild type under glucanex stress; Δ*mpkA* + glucanex/Δ*mpkA*– fold change between Δ*mpkA* under glucanex stress and Δ*mpkA* without stress.

We performed a global evaluation of genes that are regulated by MpkA through a large-scale analysis of gene expression in wild-type and Δ*mpkA* strains using a microarray hybridization approach. Previous results revealed that cell wall-damaging compounds trigger the MpkA-regulated cell wall integrity pathway (Valiante *et al*., 2008). Of all the cell wall stressors tested, glucanex, which hydrolyses the β-1,3 and β-1,6 glucan network, rapidly activated MpkA by dual phosphorylation (Valiante *et al*., 2009). To characterize how *A. fumigatus* wild-type and Δ*mpkA* strains respond and adapt to glucanex-induced cell wall perturbation, the transcriptome after exposure to glucanex was studied. In all the comparisons, 608 genes were differentially expressed, i.e. either induced or repressed at least 1.5-fold. An overview of genes that attribute new functions to MpkA and connect the MpkA pathway to previously unrelated processes in *A. fumigatus* is presented (Fig. 1 and Figs S1 and S2). For selected genes expression patterns identified by transcriptome analysis were confirmed either by Northern blot analysis or by tests at the protein level.

### Cell wall-related genes

**Fig. 2:**
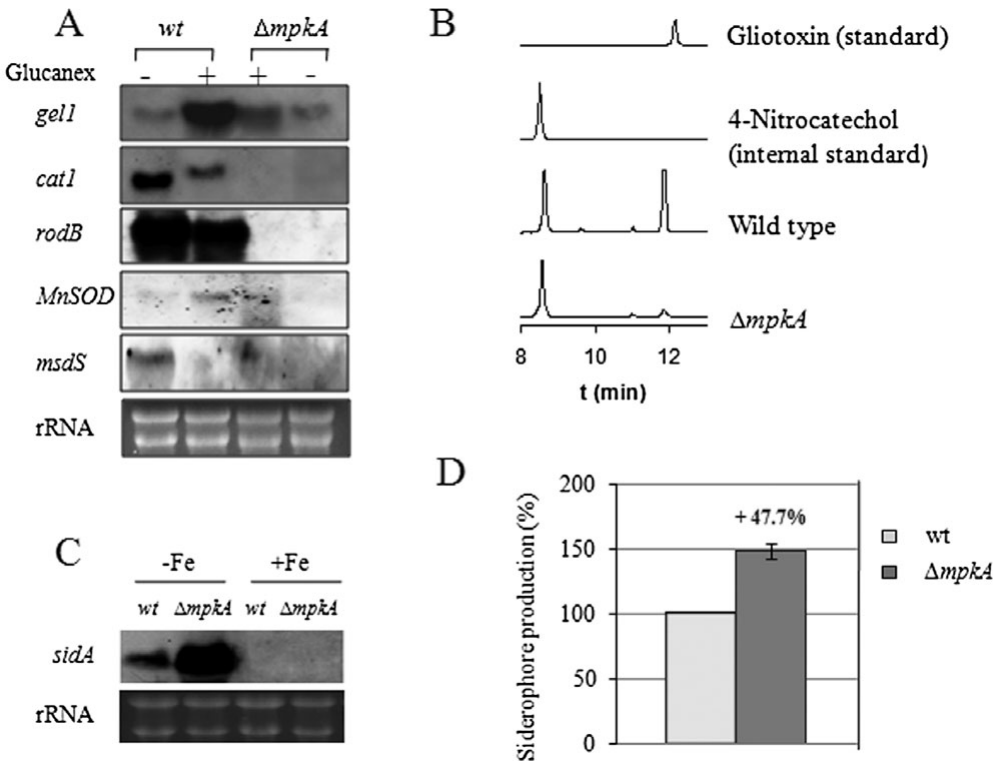
A. Validation of microarray data by Northern blot analysis. Transcripts of the indicated genes were monitored in *A. fumigatus* wild type and Δ*mpkA* with and without addition of glucanex. B. HPLC analysis to determine gliotoxin level in the Δ*mpkA* strain compared with the wild type. C. Iron-regulated expression of *sidA* in the wild type and in strain Δ*mpkA*. For Northern blot analysis, total RNA was isolated from strains grown under +Fe and −Fe conditions. D. CAS assay showing increased level of secreted siderophores in Δ*mpkA* under iron-deficient conditions. The strains were cultivated for 14 h and the supernatants were analysed for siderophore content. The siderophore content in the wild type was normalized to 100%.

Although MpkA plays a key role in cell wall signalling in *A. fumigatus*, only a few known or predicted genes that encode proteins involved in the cell wall formation were found to be differentially regulated either in absence of *mpkA* or by activation of MpkA by glucanex. Gel1p, a GPI-anchored glucanosyltransferase, is required for elongation of β-1,3 glucan of the cell wall (Mouyna *et al*., 2000). Microarray analysis showed upregulation of the *gel1* gene by glucanex in the wild type whereas in Δ*mpkA* it was downregulated under the same conditions. However, there was no major difference in the transcript level of *gel1* when non-induced wild-type and Δ*mpkA* strains were compared. Such expression patterns suggest that contribution of Gel1 to strengthen cell-wall assembly under glucanex stress is partially regulated by MpkA. This result was further confirmed by Northern blot analysis, as shown in Fig. 2A. Interestingly, another predicted GPI-anchored protein with modulated expression was aspartic endopeptidase Yap2. Its orthologues in *Saccharomyces cerevisiae* and *Candida albicans* have been implicated in maintaining cell-wall assembly by regulating β-glucan synthesis (Krysan *et al*., 2005; Albrecht *et al*., 2006). Although the role of *yap2* in *A. fumigatus* has yet to be determined, the transcriptional profile revealed that it is induced in the Δ*mpkA* mutant under both cell wall stress and non-stress conditions. Furthermore, β-glucanase, an enzyme also involved in synthesis of β-1,3 glucan (Gastebois *et al*., 2009), showed similar upregulation in the Δ*mpkA* strain. While genes involved in hydrolysing β-1,3 glucan showed enhanced expression in the mutant, the α-1,3 glucanase gene (Gastebois *et al*., 2009) required for modification of α-1,3 glucan displayed reduction in the mutant under non-cell wall stress conditions. Other genes that showed downregulation in the Δ*mpkA* strain were *rodB*, a hydrophobin playing a role in maintaining the structure of the conidial cell wall (Paris *et al*., 2003a), mannosidase *msdS* (Li *et al*., 2008) and dimethylallyl tryptophan synthase. The latter two are both related to *N*-glycan processing and thus affect the organization of the fungal cell wall (Fig. 2A).

### Genes mediating protection against ROS

In *A. fumigatus*, the deletion of *mpkA* resulted in an increased sensitivity to menadione and diamide, but an enhanced tolerance towards H_2_O_2_ (Valiante *et al*., 2008). Therefore, it was conceivable to find genes for protection against ROS to be differentially expressed. Catalases are extracellular antioxidants which protect cells against oxidative damage caused by hydrogen peroxide. The *catA* gene which encodes the conidial catalase (Paris *et al*., 2003b) was highly upregulated in Δ*mpkA* in comparison with the wild type. On the other hand, *cat1* encoding the mycelial catalase was downregulated in the mutant, as also verified by Northern blot analysis (Fig. 2A). Likewise, mitochondrial MnSOD (Asp f6), a hyphae-specific metalloenzyme causing dismutation of the toxic superoxide anion (Schwienbacher *et al*., 2005; Lambou *et al*., 2010), had reduced transcript levels in Δ*mpkA* under standard conditions, whereas MnSOD showed an increased expression in the glucanex-challenged Δ*mpkA* strain (Fig. 2A). Interestingly, *pes1*, a non-ribosomal peptide synthetase (NRPS)-encoding gene which contributes to tolerance of *A. fumigatus* against oxidative stress (Reeves *et al*., 2006), was also found to be downregulated in the Δ*mpkA* mutant. In contrast, cell wall stress results in upregulation of *pes1* in the wild type when compared with non-stressed conditions.

### Secondary metabolism genes

*Aspergillus fumigatus* is able to produce a diverse array of natural products in the form of secondary metabolites that confer some benefits to the organism while also acting as virulence determinants. Interestingly, transcriptome analysis revealed that several genes belonging to different secondary metabolite gene clusters displayed altered expression (Fig. S3). Genes corresponding to the pyomelanin cluster, such as *hmgA*, *fahA*, *maiA*, were all upregulated in Δ*mpkA* especially when cell wall stress was applied. Pyomelanin is a melanin produced in *A. fumigatus* via the l-tyrosine degradation pathway (Schmaler-Ripcke *et al*., 2009). It was shown that pyomelanin formation is affected by cell wall stress (Valiante *et al*., 2009). In contrast, several genes belonging to the gliotoxin gene cluster of *A. fumigatus* were downregulated. Gliotoxin is produced by almost all clinical *A. fumigatus* isolates (Kupfahl *et al*., 2008; Scharf *et al*., 2010). Genes coding for GliN, a putative methyltransferase, and GliT, the thioredoxin reductase in the gliotoxin cluster, showed up to threefold reduction in mRNA level in the mutant compared with the wild type. To verify the transcriptome data at the product level, the amount of gliotoxin produced in the Δ*mpkA* mutant in comparison with the wild type was quantified by HPLC in Czapek-Dox medium. The mycelial dry weight of strain Δ*mpkA* cultivated in Czapek-Dox medium was only reduced by 5% in comparison with the wild type. In the wild type, the gliotoxin concentration was 14.4 µg gliotoxin per mg of fungal dry weight (SE ± 2.8 µg mg^−1^) whereas in Δ*mpkA*, gliotoxin level was reduced to 2 µg mg^−1^ (SE ± 0.1 µg mg^−1^) (Fig. 2B). These data demonstrate that MpkA plays a role in gliotoxin production in *A. fumigatus*.

One interesting outcome of the microarray analysis was the differential expression of genes involved in siderophore biosynthesis in *A. fumigatus*. Siderophores are low-molecular-weight iron-chelating ligands produced by most fungi under conditions of extreme iron stress (Neilands, 1993; Guerinot, 1994). The production of siderophores is an important virulence determinant of *A. fumigatus*. The first committed step in biosynthesis of hydroxamate siderophores is *N*(5)-hydroxylation of ornithine catalysed by the *sidA*-encoded enzyme l-ornithine-5-monooxygenase (Schrettl *et al*., 2004). Since the medium used for cultivating strains for the microarray analyses contained iron, we therefore planned to use media devoid of iron to investigate whether absence of *mpkA* in such condition had any effect on siderophore biosynthesis. In fact, under iron depletion, the steady-state mRNA level of *sidA* was twofold increased in the Δ*mpkA* strain in comparison with the wild type (Fig. 2C). This increase in *sidA* transcript level in Δ*mpkA* led us to investigate whether a corresponding increase in the amount of secreted siderophores in the Δ*mpkA* mutant occurred. As shown in Fig. 2D, a 1.5-fold increase in siderophore level was detectable in the Δ*mpkA* strain in comparison with the wild type. This finding points to the possible involvement of *mpkA* in siderophore production in *A. fumigatus* under iron limitation.

### Iron starvation triggers MpkA activation and localization

**Fig. 3:**
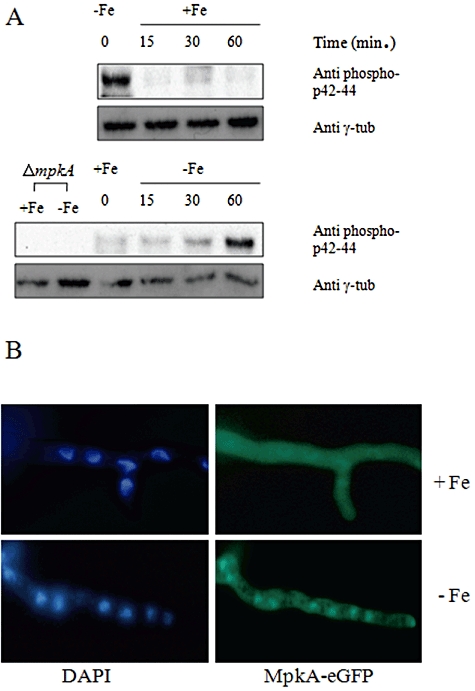
A. Western blot analysis to determine activation by phosphorylation of MpkA under iron starvation conditions. The wild type was pre-cultivated in iron-depleted medium. FeSO_4_ (10 µM) was added and samples were collected at the indicated time points (upper panel). For reverse experiment, wild type was cultivated in iron-sufficient medium before adding 1 mM desferrioxamin. Samples were collected at indicated time points (lower panel). The phosphorylated MpkA was detected by using anti-phospho p42-44 antibody. As a negative control, Δ*mpkA* strain under −Fe and +Fe condition was used which showed no phosphorylation band. Total protein was detected by immunoblotting with γ tubulin antibody as loading control. B. Microscopic pictures for detection of MpkA–eGFP in the nucleus under −Fe conditions. Nuclear stain used was 4′,6-diamidino-2-phenylindole (DAPI).

To investigate the role of MpkA in siderophore production in more detail, it was of interest to test whether iron starvation functions as signal for activation of the MpkA-related MAPK module. For this purpose, Western blot analysis was carried out to analyse the MpkA phosphorylation status in the wild-type strain under iron-depletion and iron-repletion conditions. The wild-type strain was grown in iron-depleted AMM overnight and samples were collected before and at different time points after addition of iron. Under iron-depleted conditions, MpkA is present in the phosphorylated form. Directly after addition of 10 µM FeSO_4_ a strong decrease in the amount of phosphorylated MpkA is detected at time points 15–60 min. To further evaluate these data, an inverse experiment was performed wherein 1 mM desferrioxamin (DFO) was used as an iron chelator. The wild-type strain was grown in iron-sufficient AMM overnight and samples were collected before and at different time points after addition of DFO. Under iron-sufficient conditions, MpkA showed basal level of phosphorylation. After addition of DFO the phosphorylation of MpkA increased after 60 min showing that MpkA was activated in absence of iron (Fig. 3A). These results support the hypothesis that iron starvation activates MpkA signalling in *A. fumigatus*. Furthermore, to gain insight into the expression and localization of MpkA under iron deprivation, an *mpkA–egfp* fusion under the control of a constitutive promoter was generated. This gene fusion was introduced in both the wild type and the Δ*mpkA* mutant strain. Remarkably, transformants of Δ*mpkA* with an ectopic integration of the *mpkA–egfp* fusion construct showed wild-type growth, confirming that the MpkA–eGFP fusion protein is functional (Fig. S4). An eGFP signal was visible in all transformants. During iron-replete conditions, fluorescence was observed to be distributed evenly throughout the cell. During iron starvation conditions, however, fluorescence decreased in the cytoplasm and concentrated in the nuclei (Fig. 3B). Interestingly, exposing *A. fumigatus* to cell wall stress by glucanex or oxidative stress by hydrogen peroxide led to accumulation of MpkA–eGFP in the nucleus (Fig. S5).

### MpkA inhibits siderophore biosynthesis in a HapX/SreA-independent manner

*Aspergillus fumigatus* secretes siderophores for iron acquisition and possesses intracellular siderophores for iron storage. During iron sufficiency, the GATA transcription factor SreA operates as a negative regulator by repressing iron acquisition and siderophore biosynthesis whereas the bZIP transcription factor HapX mediates repression of iron-dependent pathways and activation of siderophore biosynthesis during iron starvation (Hortschansky *et al*., 2007; Schrettl *et al*., 2008; 2010a). Mutual transcriptional control maintains a tight interplay between HapX and SreA, as HapX represses the expression of *sreA* under iron limitation while availability of iron represses expression of *hapX* via SreA

**Fig. 4:**
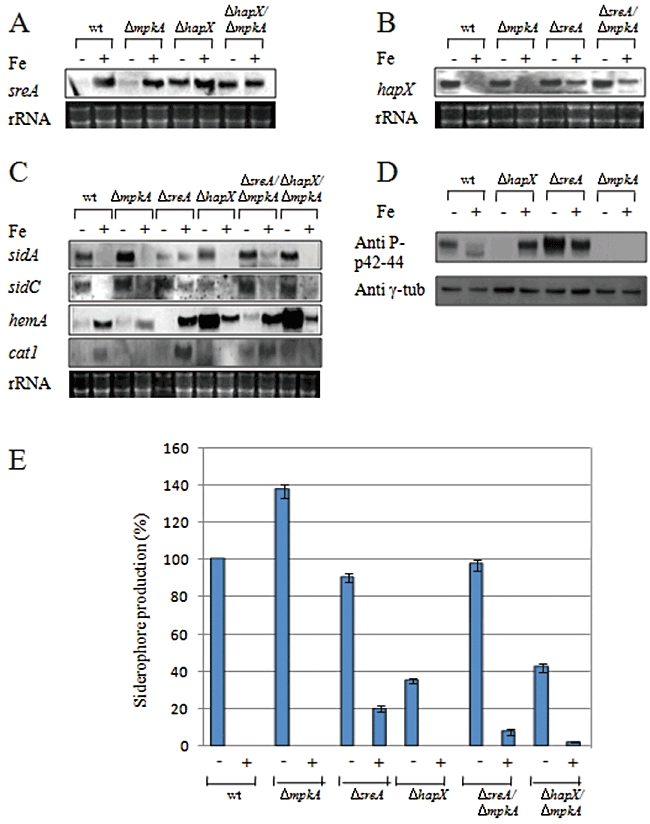
A–C. Iron-regulated mRNA levels of iron acquiring and iron-dependent genes in *A. fumigatus* wild-type, Δ*mpkA*, Δ*sreA*, Δ*hapX*, Δ*sreA/*Δ*mpkA* and Δ*hapX/*Δ*mpkA* strains. rRNAs are shown as loading control. D. Western blot analysis to determine activation by phosphorylation of MpkA in *A. fumigatus* wild-type, Δ*sreA* and Δ*hapX* strains. The γ tubulin antibody was used as loading control. E. CAS assay for quantification of secreted siderophores in different *A. fumigatus* strains under −Fe and +Fe conditions.

Because MpkA appears to reduce siderophore biosynthesis in *A. fumigatus*, we investigated whether this MpkA-dependent inhibition of siderophore requires HapX or SreA. Therefore, we generated mutants lacking *hapX* or *sreA* in a Δ*mpkA* background (Fig. S6). Northern blot analysis revealed that the Δ*mpkA* mutant and the wild type maintained equal levels of *sreA* transcript under iron availability. It is interesting to note that expression of *sreA* also remained unaltered in the Δ*hapX/*Δ*mpkA* mutant compared with the Δ*hapX* single mutant under iron limitation (Fig. 4A). This finding indicates that MpkA plays no role in regulating *sreA* levels under any of the aforementioned conditions and that the expression of *sreA* during iron deprivation is due to impairment of transcriptional regulation by HapX on *sreA*. Likewise, mRNA levels of *hapX* were unaffected in wild type, Δ*mpkA*, Δ*sreA* and Δ*sreA/*Δ*mpkA* under iron limitation and also in Δ*sreA* and Δ*sreA/*Δ*mpkA* under iron-replete conditions (Fig. 4B). Hence, *hapX* is not regulated by MpkA at the transcript level. These findings imply that expression of *hapX* and *sreA* is *mpkA*-independent.

Previous studies showed that deficiency in *hapX* or *sreA* does not affect the transcript level of *sidA* under iron limitation (Hortschansky *et al*., 2007; Schrettl *et al*., 2008; 2010a) (Fig. 4C). However, deficiency in *hapX*, but not *sreA*, substantially decreased extracellular production of siderophores. Our data show that under −Fe conditions, deficiency of *mpkA* led to an increase in both *sidA* transcript level and extracellular siderophore production (Fig. 4C and E). Moreover, deficiency in *mpkA* increased the *sidA* transcript level in both Δ*hapX* and Δ*sreA* backgrounds, paralleled by a slight increase in extracellular siderophore production (Fig. 4C and E). During iron starvation, the lack of *sreA* did not significantly alter the level of *sidA*, and this is also reflected at the siderophore level (Schrettl *et al*., 2008). These data imply that under iron starvation MpkA activation leads to a decrease of siderophore production independently of SreA and HapX.

Furthermore, Northern blot analyses indicated that during iron starvation MpkA is not involved in the regulation of genes related to iron-consuming pathways such as the haem-containing catalase *cat1* and *hemA* encoding 5-aminolevulinate synthase. The latter catalyses the first committed step in haem biosynthesis (Fig. 4C). Under standard Fe conditions these two genes have reduced expression in Δ*mpkA*, particularly *cat1*, as shown in Figs 2A and 4C. In the Δ*sreA/*Δ*mpkA* mutant under −Fe at least low expression of these genes is observed in comparison with no detectable expression in the Δ*sreA* mutant. This slight increase in expression appears to be a rather indirect effect due to iron accumulation, as iron can be taken up by increased production of siderophores due to deficiency of the *mpkA* gene. These data further underline that the influence of MpkA on siderophore biosynthesis is independent of HapX and SreA.

During iron depletion, we observed a low phosphorylation status of MpkA in the Δ*hapX* mutant strain, whereas MpkA was highly phosphorylated in the Δ*sreA* strain (Fig. 4D). These data confirmed that during iron depletion MpkA is acting in response to siderophore production. Additionally, during normal growth conditions, MpkA phosphorylation status was always higher in the Δ*sreA* and Δ*hapX* mutant strains compared with the wild type. These data suggested that deletion of *sreA* and *hapX* affected to a certain extend the cell wall integrity pathway. The increased sensitivity of the Δ*sreA* and Δ*hapX* mutant strains against Congo red confirmed this observation (Fig. S7).

### MpkA is required for adaptation to iron starvation

**Fig. 5:**
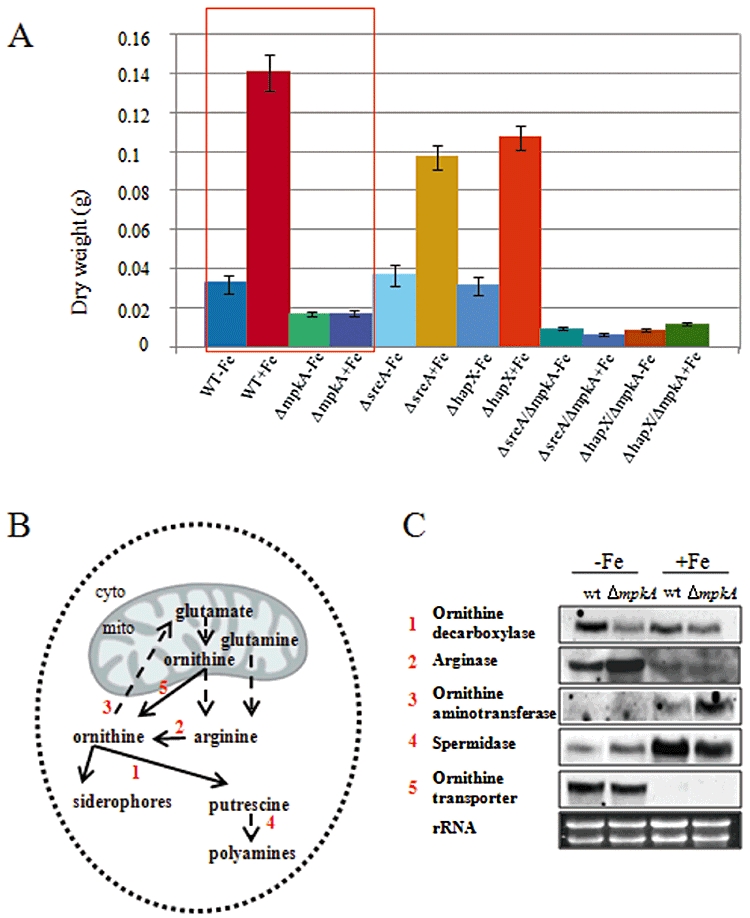
A. Dry weight of *A. fumigatus* wild type, Δ*mpkA*, Δ*sreA*, Δ*hapX*, Δ*sreA/*Δ*mpkA* and Δ*hapX/*Δ*mpkA* grown in −Fe and +Fe conditions. B. Schematic representation of ornithine metabolism in mitochondria (mito) and cytoplasm (cyto) of *A. fumigatus*. Ornithine is synthesized from glutamate in mitochondria or from arginine in cytoplasm. Mitochondrial ornithine is transported to the cytoplasm via an ornithine transporter. Ornithine acts as a precursor for both siderophore and polyamine biosynthesis. The enzymatic steps involved in these conversions are numbered and corresponding Northern blot analysis is presented in (C). C. Northern blot analysis showing differential expression of genes involved in ornithine and polyamine biosynthesis in the wild type and in Δ*mpkA* grown in −Fe and +Fe conditions. rRNAs are shown as loading control. Accession numbers of the genes are as follows: Ornithine aminotransferase Afu4g09140, Spermidase Afu1g13490, Arginase Afu3g11430, Ornithine transporter Afu8g02760, Ornithine decarboxylase Afu4g08010.

The mechanisms by which MpkA is able to reduce secretion of siderophores under Fe starvation were further studied. Inhibition of siderophore biosynthesis via MpkA seems unnecessary under iron limitation. When the dry weights of the wild-type and Δ*mpkA* strains grown overnight in Fe-rich and Fe-deficient medium were compared, the dry weight of the wild type grown in −Fe medium was significantly reduced in comparison with the wild type grown in +Fe medium (Fig. 5A). This was expected, as Fe starvation reduces the general growth rate of the fungus. Surprisingly, the dry weights of Δ*mpkA* strains grown in Fe-rich and Fe-deficient medium were similar (Fig. 5A), indicating that the Δ*mpkA* strain does not adapt its growth rate to iron limitation.

Recent data have shed light on iron starvation-driven remodelling of the free amino acid pool in *A. fumigatus* wherein highlighting the importance of upregulation of the cellular ornithine pool as a precursor to stimulate siderophore biosynthesis (Schrettl *et al*., 2010a,b;) (Fig. 5B). Moreover, *hapX* was shown to be essential for this metabolic co-ordination. With the result of dry weight measurement as a key indicator of modified yet comparatively robust metabolism in Δ*mpkA* strain, we hypothesized a reshuffle of the amino acid pool occurred, particularly of ornithine, under iron-limitation and iron-replete conditions in these mutants. The wild type and Δ*mpkA* mutant strain were grown in iron-depleted and iron-replete media. The samples were subsequently subjected to HPLC analysis for determining the concentration of free amino acids (Table 1). The total quantity of free amino acids extracted from Δ*mpkA* was always lower than the quantity extracted from the wild type. Ornithine is synthesized either from glutamate or from ornithine-derived arginine (Fig. 5B). The arginine level showed a 3.2-fold increase in the wild type grown under −Fe conditions compared with +Fe conditions and an increase of 4.5-fold was observed in strain Δ*mpkA* cultivated under −Fe conditions versus Δ*mpkA* cultivated under +Fe conditions (Table 1). Glutamine, which is a precursor for arginine, showed a 14.2-fold increase in Δ*mpkA* under −Fe conditions, while in the wild type the fold change was 5.6. Glutamate levels remained constant in the wild type and in the Δ*mpkA* strain under +Fe and −Fe. In strain Δ*mpkA*, the ornithine level increased ninefold under Fe deplete conditions, whereas in the wild type only an increase of 2.6-fold occurred. In general, in the Δ*mpkA* mutant a high level of free ornithine during iron-depletion condition was present. Ornithine is also converted into polyamines which are important for the growth and development of all cells. Polyamines consist of the diamine putrescine, the triamine spermidine and the tetraamine spermine. Polyamine measurements showed that putrescine and spermidine levels decreased in the wild type during iron depletion, while they remained constant in the Δ*mpkA* mutant (Table 1). On the contrary, the level of spermine remained quite constant in the wild type while it increased in the mutant. Furthermore, Northern blot analysis showed upregulation of an arginase gene in Δ*mpkA* in comparison with the wild type under −Fe conditions (Fig. 5C). Arginase catalyses the conversion of arginine into ornithine which can be further used as a precursor for siderophores, polyamines and cytoplasmic glutamate (Schrettl *et al*., 2010a). Strikingly, ornithine decarboxylase, which catalyses conversion of ornithine to polyamines via putrescine, was downregulated in Δ*mpkA* under iron deficiency. This result in conjunction with the increase in glutamine levels in the Δ*mpkA* strain under iron starvation implied that arginine was continuously converted into ornithine to fuel siderophore biosynthesis. Since ornithine aminotransferase, responsible for recycling ornithine back to the glutamate cycle, showed no change in expression and the glutamate level decreased in the mutant under iron starvation, these data indicated that ornithine was not converted back to glutamate. Moreover, both the ornithine transporter (AmcA) required for transport of ornithine from mitochondria to the cytoplasm and spermidine synthase showed no change in expression in the Δ*mpkA* mutant as well. It appears that the Δ*mpkA* mutant responds to iron starvation by using increased level of free ornithine for siderophore biosynthesis without exhibiting any negative effect on cellular processes. These data indicate that MpkA is required for adaptation to iron starvation by reshuffling of the pool of amino acids.

**Table 1:** Change of amino acid pool and polyamine composition in *A. fumigatus* during iron starvation (µmol g^−1^ dry weight)

aa	+Fe wt	−Fe wt	−Fe/+Fe wt	+Fe Δ*mpkA*	−Fe Δ*mpkA*	−Fe/+Fe Δ*mpkA*
Arg	204.9 ± 42.9	645.7 ± 182.93	3.1	102.7 ± 22.1	458.3 ± 13.6	4.5
Asn	1218.4 ± 383.6	817.7 ± 257.14	0.7	226.0 ± 38.5	215.1 ± 43.4	0.9
Asp	27.8 ± 3.7	21.8 ± 11.4	0.8	11.3 ± 1.8	14.5 ± 0.8	1.3
Gln	148.7 ± 40.8	834.3 ± 229.0	5.6	34.5 ± 5.3	491.5 ± 5.9	14.2
Glu	419.4 ± 112.2	375.6 ± 100.1	0.9	118.8 ± 14.8	131.2 ± 8.1	1.1
His	17.5 ± 1.8	120.3 ± 39.2	6.8	10.1 ± 2.0	85.0 ± 5.9	8.4
Ile	68.2 ± 9.0	81.2 ± 22.8	1.2	16.2 ± 5.3	77.9 ± 0.5	4.8
Leu	88.8 ± 13.3	131.5 ± 40.2	1.5	25.3 ± 7.5	106.8 ± 2.3	4.2
Lys	125.3 ± 35.7	496.9 ± 220.8	3.9	58.1 ± 15.9	278.6 ± 5.9	4.8
Orn	230.4 ± 62.6	590.0 ± 176.5	2.5	63.2 ± 21.2	575.6 ± 36.2	9.1
Phe	53.6 ± 12.1	96.4 ± 27.2	1.8	25.5 ± 8.1	59.8 ± 1.7	2.3
Ser	304.1 ± 82.6	306.6 ± 102.3	1.0	50.8 ± 12.1	216.9 ± 7.0	4.3
Thr	194.0 ± 45.7	163.7 ± 43.0	0.8	43.4 ± 13.9	131.5 ± 34.3	3.0
Trp	49.5 ± 10.4	29.3 ± 10.2	0.6	12.5 ± 4.0	31.6 ± 1.0	2.5
Tyr	1415.9 ± 365.7	811.5 ± 88.1	0.6	249.5 ± 64.7	814.9 ± 173.1	3.3
Val	236.3 ± 50.8	180.3 ± 47.9	0.7	48.9 ± 13.6	160.5 ± 4.5	3.3
Polyamines						
Putrescine	9.7 ± 1.0	5.7 ± 0.4	0.6	7.4 ± 2.5	6.1 ± 1.8	0.8
Spermidine	143.8 ± 18.9	86.5 ± 13.6	0.6	62.9 ± 17.3	61.8 ± 5.6	0.9
Spermine	28.7 ± 2.8	23.6 ± 3.2	0.8	7.5 ± 1.8	20.8 ± 1.5	2.7

## Discussion

The aim of this study was to get a deeper insight into the role of the cell wall integrity signalling pathway in *A. fumigatus*. Microarray analyses carried out here demonstrated that MpkA regulates genes involved in maintaining the cell-wall assembly indicating a role of MpkA in restructuring the cell wall by differentially regulating enzymes involved in either synthesis or hydrolysis of cell wall glucans. In *S. cerevisiae*, a genome-wide survey of changes in gene expression resulting from activation of Mpk1/Slt2 identified 25 genes whose regulation was altered. Most of these genes encoded either known or putative proteins or enzymes involved in cell wall biogenesis. Only six genes with no obvious link to the cell wall were regulated in response to cell wall integrity signalling, one of them being cytosolic catalase T (Jung and Levin, 1999).

Our results revealed that in *A. fumigatus*, MpkA regulates the expression of both mycelial (Cat1) and conidial (CatA) catalases. In fact, induction of conidial catalase in Δ*mpkA* might explain the resistance of the Δ*mpkA* strain against H_2_O_2_, as an increased secretion of CatA augments detoxification of H_2_O_2_ in the medium (Valiante *et al*., 2008). Furthermore, reduced expression of MnSOD in absence of *mpkA* suggested that this gene is partially regulated by MpkA under standard growth conditions while activation of MpkA resulted in increased expression of MnSOD. It is thus likely that under cell wall stress, also MpkA-independent signalling controls regulation of MnSOD. Nuclear accumulation of MpkA during cell wall and H_2_O_2_ stress corroborates this hypothesis. Consistently, microarray analysis revealed that many genes in *A. fumigatus* pertaining to ROS stress are directly or indirectly regulated by MpkA supporting the hypothesis that MpkA plays a wider role in *A. fumigatus* than merely controlling cell wall repair mechanisms.

The *Schizosaccharomyces pombe* Spc1/Sty1 (Buck *et al*., 2001), *C. albicans* CaHog1 (Alonso-Monge *et al*., 2003) and *Aspergillus nidulans* SakA (Kawasaki *et al*., 2002) have been shown to be activated by ROS and to mediate oxidative stress responses. In *A. fumigatus*, apart from MpkA, mutants lacking the MAP kinase SakA have also been shown to be sensitive to certain types of ROS stress (Du *et al*., 2006). As a direct conclusion, it becomes evident that many signalling pathways, including the MpkA-regulated cell wall integrity pathway, cooperate to activate genes whose activity is required for appropriate responses against oxidative stress.

A remarkable result of the transcriptome analysis of Δ*mpkA* was the finding of differential regulation of genes involved in biosynthesis of several secondary metabolite gene clusters. Several levels of genetic regulation govern when and where secondary metabolites will be produced (Hoffmeister and Keller, 2007; Brakhage and Schroeckh, 2011). Global transcription factors involved in nitrogen (AreA), carbon (CreA), pH (PacC) homeostasis, developmentally regulated transcription factors such as StuA or the CCAAT binding factor AnCF often affect the expression of secondary metabolite gene clusters (Keller *et al*., 2005; Brakhage *et al*., 2009; Twumasi-Boateng *et al*., 2009; Brakhage and Schroeckh, 2011). Moreover, LaeA, a putative methyltransferase, is known to regulate secondary metabolism gene clusters in *Aspergilli* (Bok and Keller, 2004). Most of the work devoted to dissecting signal transduction mechanisms involved in regulating secondary metabolism in *Aspergilli* points towards involvement of G protein-coupled receptor (GPCR) signalling pathways (Hicks *et al*., 1997; Brodhagen and Keller, 2006; Gehrke *et al*., 2010). Downstream effectors of GPCRs are, e.g. adenylyl cyclase/cAMP/PKA pathways and MAP kinase pathways. The cAMP/PKA pathway has been shown to be involved in mycotoxin and melanin production in *Aspergilli* (Brodhagen and Keller, 2006; Grosse *et al*., 2008). There have not been many reports linking MAP kinases to the production of natural products in fungi. In *A. nidulans*, MAP kinase MpkB, a component of the putative FUS3 signalling pathway, regulated transcription of genes involved in secondary metabolism by partially influencing regulation of *laeA* transcription (Atoui *et al*., 2008). Previously, we showed that cell wall stress resulting in hyperphosphorylation of MpkA, increased production of the dark brownish pigment pyomelanin. The precise role of pyomelanin is unknown, but it has been proposed that it acts as a part of salvage pathway against cell wall perturbation in *A. fumigatus* (Schmaler-Ripcke *et al*., 2009; Valiante *et al*., 2009). The microarray data shown here corroborated this finding as under cell wall stress, many genes of the pyomelanin cluster were upregulated. Furthermore, HPLC analysis showed that *mpkA* deletion in *A. fumigatus* led to a decrease in production of gliotoxin, a potent immunosuppressant belonging to the epipolythiodioxopiperazine class of fungal toxins (Kupfahl *et al*., 2008; Scharf *et al*., 2010). Microarray analysis revealed that the mRNA level of *gliT*, a gene of the gliotoxin biosynthesis cluster, was significantly reduced in Δ*mpkA*. Previous results showed that deletion of *gliT* renders *A. fumigatus* highly sensitive to gliotoxin. Moreover, gliotoxin was reported to be involved in protecting *A. fumigatus* against oxidative stress which is in concordance to our results elucidating a role of MpkA in defence against ROS (Scharf *et al*., 2010; Schrettl *et al*., 2010b).

Furthermore, unexpectedly, we established a link between MpkA and production of siderophores. Siderophores are a class of small molecules that are secreted by microbes to chelate iron, and thus mediate uptake of this essential trace element. *A. fumigatus* produces two major siderophores to capture extracellular iron, namely intracellular fusarinine C (FC) and its acetylated derivative, i.e. the extracellular triacetylfusarinine C (TAFC) (Schrettl *et al*., 2007). These compounds are assembled by NRPS systems whose expression is strictly regulated by the availability of iron. A lack of both intra- and extracellular siderophores due to deficiency in the ornithine monooxygenase SidA (Δ*sidA* strain) or the iron regulator HapX (Δ*hapX* strain) was shown to render *A. fumigatus* avirulent in a neutropenic mouse model of invasive aspergillosis (Schrettl *et al*., 2004; 2010a). Here, we showed that under iron deficiency MpkA is activated by phosphorylation and localized to the nucleus. Deletion of *mpkA* increased extracellular siderophores by enhancing *sidA* expression in *A. fumigatus*. Moreover, the MpkA regulation of siderophores was independent of HapX/SreA-regulated mechanisms that were shown to be the major regulators for maintaining iron homeostasis. This is a paradox to the role siderophores play, as they ensure an adequate supply of iron for the fungus, and are of importance for fungal growth in particular during iron starvation.

The major substrate required for the formation of siderophores is ornithine (Schrettl *et al*., 2010a). Ornithine is also a precursor for polyamines in the fungal cell. The three most commonly occurring natural polyamines are putrescine, spermidine and spermine. In fungi, these compounds have been reported to be important for cell differentiation processes including sporulation, spore germination and dimorphic transition (Guevara-Olvera *et al*., 1993; Lopez *et al*., 1997). In *A. nidulans*, spermidine is essential for viability and it was suggested that certain threshold levels of spermidine are required for specific developmental processes (Jin *et al*., 2002). By analysing the amino acid pool composition of Δ*mpkA* and the wild type under iron starvation conditions, it was observed that most amino acids showing a change in the wild type also showed a change in the mutant. Although the individual amounts of amino acids were lower in the mutant than the wild type, ornithine showed a comparable level to that of the wild type under iron starvation. Increasing mRNA steady-state levels of arginase and ornithine decarboxylase in the wild type indicate that increased amounts of ornithine were preferentially used for siderophore biosynthesis than for polyamine production. In addition, the polyamine level in the wild-type strain decreased during iron starvation while it remained constant in the Δ*mpkA* mutant. The importance of polyamines in physiology is illustrated by the changes in polyamine concentration on exposure to stress conditions (Minguet *et al*., 2008). This implies that under iron deprivation MpkA competes to divert available ornithine in production of polyamines for essential survival processes. Remarkably, in contrast to the wild type the Δ*mpkA* mutant strain showed no difference in biomass production under iron-deficient and iron-sufficient conditions. In contrast, the wild type grown in iron-deficient medium showed a significantly reduced dry weight compared with growth in iron-sufficient medium. This finding suggests that Δ*mpkA* did not react in response to the lack of iron.

**Fig. 6:**
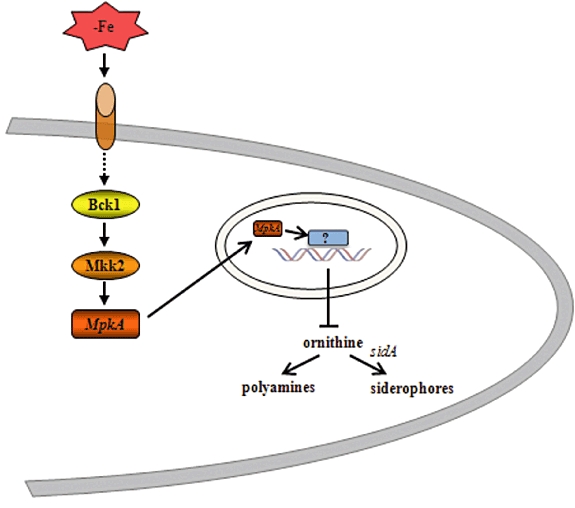
Model depicting inhibition of siderophore biosynthesis by MpkA under iron starvation in *A. fumigatus*. Under conditions of iron starvation MpkA is activated and localized to the nucleus. A role of MpkA in the nucleus is to activate unknown transcription factor/factors (?) leading to overall decrease in siderophore biosynthesis by reduction of ornithine (precursor for siderophore biosynthesis) and by downregulation of the *sidA* gene.

From these data, a mechanism by which MpkA inhibits siderophore biosynthesis is suggested: MpkA acts as a repressor of ornithine synthesis thereby depleting the main substrate for siderophore biosynthesis as well as it affects the siderophore production at the gene level by dowregulating *sidA* expression. It will be very interesting to identify MpkA-regulated transcription factors which affect the above-mentioned phenomena (Fig. 6). We propose, MpkA is able to fine-tune the level of siderophore biosynthesis to save cellular energy under iron-depleted conditions and thus improves survival.

Taken together, we show that in *A. fumigatus*, MpkA-regulated cell wall integrity signalling is involved in regulation of a plethora of genes ranging from those involved in cell wall repair and synthesis, defence against oxidative stress, pigment and toxin biosynthesis as well as siderophore production. MpkA appears to fine-tune the balance between the stress responses and the energy consumed in cellular processes required for growth, development and secondary metabolite biosynthesis.

## Experimental procedures

### Fungal and bacterial strains, media and growth conditions

*Aspergillus fumigatus* strains used in this study are listed in Table 2. Strains were grown in *Aspergillus* minimal medium (AMM) as described previously (Weidner *et al*., 1998). AMM agar was prepared by addition of 1.6% (w/v) Select Agar (Invitrogen, Germany). If required, pyrithiamine (Sigma Aldrich, Germany) or hygromycin (Roche Applied Science, Germany) in a final concentration of 0.1 µg ml^−1^ and 200 µg ml^−1^, respectively, were added. For studies on iron regulation, fungal strains were grown at 37°C in AMM, containing 1% (w/v) glucose as carbon source, 20 mM glutamine as nitrogen source, trace elements according to Cove including 30 µM FeSO_4_ as iron source (+Fe conditions). Trace elements without iron were used for −Fe conditions. For transformation of *Escherichia coli*, TOP10F′ cells (Invitrogen, Germany) were used. *E. coli* cells were grown at 37°C in LB medium supplemented with 100 µg ml^−1^ ampicillin or 50 µg ml^−1^ kanamycin when required.

**Table 2:** Fungal strains used in this study

Strain	Description	Reference
CEA17Δ*akuB*^KU80^	*akuB*^KU80^::*pyrG*; PyrG^+^	da Silva Ferreira *et al*. (2006)
Δ*mpkA*	*mpkA*::*ptrA*; Δ*mpkA*, Δ*akuB*, PT^R^	Valiante *et al*. (2009)
Δ*hapX*	*hapX*::*hph*; Δ*hapX*, Hyg^R^	Schrettl *et al*. (2010a,b);
Δ*sreA*	*sreA*::*hph*; Δ*sreA*, Hyg^R^	Schrettl *et al*. (2008)
Δ*hapX/*Δ*mpkA*	*hapX*::*hph*, *mpkA*::*ptrA*; Δ*hapX,*Δ*mpkA*, PT^R^, Hyg^R^	This study
Δ*sreA/*Δ*mpkA*	*sreA*::*hph*, *mpkA*::*ptrA*; Δ*sreA,*Δ*mpkA*, PT^R^, Hyg^R^	This study
*otef*p–*mpkA–egfp*	ATCC46645 derivative; *otef*p–*mpkA–egfp*; Hyg^R^	This study
*otef*p–*mpkA–egfp* (comp)	Δ*mpkA* derivative; *otef*p–*mpkA–egfp*; Hyg^R^, MpkA^+^	This study

### Generation of recombinant *A. fumigatus* strains

Primers used in this study are listed in Table S1. For the generation of plasmid *otef*p–*mpkA–egfp*, the *mpkA* gene was amplified by PCR from plasmid pCR2.1mpkA (Valiante *et al*., 2008) using the oligonucleotides MpkA_for_1 and MpkA_SmaI_rev. The resulting DNA fragment was cut with SmaI and BamHI, then inserted into the BamHI and SmaI site of plasmid p123otef-hph. In addition to the *otef* promoter, this vector included the *egfp* gene and the hygromycin resistance cassette. The resulting plasmid p123otefp–mpkA–eGFP carrying the *mpkA* gene under the control of the *otef* promoter was used to transform *A. fumigatus* wild-type strain ATCC 46645. Several hygromycin-resistant transformants were obtained and analysed by fluorescence microscopy and Southern blot (data not shown). One of them, producing the MpkA–EGFP fusion protein, was designated as ATCC46645otefp–mpkA–eGFP and used for further studies. For deletion of *hapX* the ‘three fragment fusion PCR technique’ was employed. Primer pairs HapX_for_1, HapX_rev(hph) and HapX_for(hph), HapX_rev_1 were used to amplify the 5′ and 3′ flanking regions of the *hapX* gene respectively. Oligonucleotides Hph_For and Hph_Rev were used to amplify the hygromycin resistance cassette from plasmid pUChph. The HapX_rev(hph) and HapX_for(hph) primers also contained sequences complementary to the sequence of Hph_for and Hph_rev primers. In the first round of PCR, the 5′ and 3′ flanking region of the *hapX* gene and hygromycin gene were amplified separately. To fuse these three PCR products, they were purified and a fusion PCR was performed using HapX_for_1 and Hapx_rev_1 primers. Phusion high-fidelity DNA polymerase (Finnzymes, Finland) was used for all PCRs. The *A. fumigatus*Δ*mpkA* strain was transformed with the *hapX* deletion plasmid (Valiante *et al*., 2009). For obtaining the *sreA* disruption construct, primer pairs SreA_for_2 and SreA_rev_2 were used to amplify the *sreA* disruption cassette from strain Δ*sreA* (Schrettl *et al*., 2008). *A. fumigatus*Δ*mpkA* strain was also transformed with this disruption construct.

### Transcriptome analysis

Full genome transcriptomic analyses were performed at Febit (Germany) (http://www.febit.de). Each array on the biochip comprises 15 000 probes. These probes are 30-mers covering all postulated gene transcripts that were designed based on the available genome sequence. Probes were selected according to a preliminary experiment that determined probes with highest specificity. The best probe was calculated for each gene fragment of interest. The second-best probes were chosen for gene fragments longer than 1200 bp. The intensities of blank probes which only consist of one single T nucleotide are used for background corrections. Blank, labelling control and hybridization control probes are not included in the data analysis. To assess the reproducibility of the biological replicates, scatter plots were made and the Pearson correlation coefficients were calculated. For comparing two sample types, the data set of both samples was normalized using the ‘Quantile/Quantile’ method. The results of the microarray analyses were deposited in the OmniFung Data Warehouse (http://www.omnifung.hki-jena.de; see *A. fumigatus* collection ‘Glucanex stress’).

For microarray analyses, the Δ*mpkA* mutant was inoculated in a four times higher amount than the wild type to achieve equal glucose consumption for both strains (Valiante *et al*., 2009). Whole RNA was isolated from wild-type and Δ*mpkA* cultures grown for 14 h in AMM at 37°C. For the analysis of cell wall stress, RNA was isolated 20 min after addition of glucanex [0.4% (w/v), Novozyme, Denmark]. Two biological replicates for each strain under inducing and non-inducing conditions were analysed. RNA was isolated by Qiagen RNeasy Plant Mini kit (Qiagen, Germany). Additionally, the samples were treated with DNase (TurboDNA-free kit, Ambion, Germany). Raw data were analysed according to Febit's protocol.

### Fluorescence analysis

For fluorescence microscopy of mycelia, the strains were grown in 3 ml of AMM supplemented with appropriate supplements at 37°C. For all microscopic studies, a Leica DM4500 B digital fluorescence microscope (Leica Microsystems, Germany) was used. Nuclei were stained with 4′,6-diamidino-2-phenylindole (DAPI) for 1 min. For documentation, a Leica DFC480 digital camera was used.

### Western blot analysis

Conidia from *A. fumigatus* were incubated in AMM for 14 h in iron-depleted medium. FeSO_4_ (10 µM) was added. Mycelia were harvested at different time points (15, 30 and 60 min after addition of 10 µM FeSO_4_) and immediately frozen in liquid nitrogen. For reverse experiment, conidia were incubated in AMM for 14 h and then 1 mM desferrioxamin (DFO) (Sigma, Germany) was added as iron chelator. Mycelia were harvested as well at different time points (15, 30 and 60 min after addition of 1 mM DFO) and immediately frozen in liquid nitrogen. One hundred milligrams mycelium was ground under liquid nitrogen to a fine powder using mortar and pestle. Protein extraction and Western blot analysis was carried out as described before (Valiante *et al*., 2009).

### Northern blot analysis

For analysis of transcript levels of different genes during cell wall stress condition, 0.4% (w/v) glucanex was added to *A. fumigatus* wild type and Δ*mpkA* pre-cultivated for 14 h in AMM. Samples were collected at 0 min and 20 min after addition of glucanex and immediately frozen in liquid nitrogen. Iron-regulated gene expression was evaluated by isolation of RNA from cultures grown for 14 h under +Fe and −Fe conditions. Total RNA (10 µg) was separated on a denaturing agarose gel and blotted onto Hybond N^+^ nylon membranes. Probe labelling, hybridization and detection were performed using the DIG Labelling Mix, DIG Easy Hyb and the CDP-Star ready-to-use kit according to the instructions of the manufacturer (Roche Applied Science, Germany).

### CAS assay

The CAS assay was used to measure the total extracellular siderophore activity of the different strains (Schwyn and Neilands, 1987). The assay is based on the competitive exchange of iron(III) from an indicator dye, chrome azurol S (CAS). The affinity of CAS for iron(III) is slightly lower than that of most siderophores, and hence the metal ion is quantitatively released to a competing ligand. The colour of the dye changes from blue to orange which is measured by absorbance at 630 nm. The strains were grown for 14 h in +Fe and −Fe media at 37°C and the supernatants were collected by filtration while the mycelium was dried and weighed for biomass measurement. An aliquot of the supernatant was used for the CAS assay. A standard curve based on different dilutions of ferrichrome was calculated. For deducing the percentage of siderophores secreted, the CAS assay reading of each strain was divided by its dry weight (g) respectively.

### Gliotoxin measurement by HPLC

The *A. fumigatus* wild-type and Δ*mpkA* strains were cultivated in 250 ml of Czapek-Dox medium at 28°C for 7 days. For gliotoxin extraction, mycelium was separated from the culture supernatant by Miracloth. To the culture supernatant, 2.8 mg of 4-nitrocatechol was added as internal standard before extraction. Extraction was done twice with 100 ml of ethyl acetate. The combined organic phases were dried with Na_2_SO_4_, and the solvent was removed under reduced pressure. The samples were re-dissolved in 8 ml of methanol and measured on a JASCO HPLC with DAD monitoring. For HPLC measurements, 20 µl of the concentrated sample were injected. A Nucleosil 100 (250 × 4.6 mm, 5 µm) column was used at a flow rate of 1 ml min^−1^ with the following gradient: A, H_2_O, 0.1% (v/v) TFA; B, acetonitrile; start 20% B, in 20 min 65% B, after 28 min 100% B for 10 min. Gliotoxin standard elutes after 12 min and the internal standard 4-nitrocatechol elutes after 8.6 min. For quantification of gliotoxin, a calibration curve was calculated from 16 µg to 1 mg. For quantification of the internal standard, a calibration curve was calculated from 63 µg to 1 mg.

### Analysis of amino acids and polyamines

The analysis of amino acids was performed according to the procedure reported by de Kraker *et al*. (2007) with slight modifications. Briefly, samples were extracted with 0.1 N HCl, adjusted to pH 10 with 0.5 M potassium borate buffer and pre-column derivatized with *o*-phathaldehyde (OPA)/mercaptoethanol. The derivatized samples were analysed on a Hewlett Packard HP 1100 Series HPLC system with autosampler and fluorescence detector (FLD) (Ex 340 nm, Em 445 nm). The gradient was optimized to detect ornithine. A Chromolith Performance RP-18e column (4.6 mm i.d., Merck, Germany) was used at a flow rate of 1.5 ml min^−1^ at 28°C with the following gradient of 0.02 M citrate buffer of pH 5.5 (solvent A) and methanol:acetonitrile (65:35, v : v; solvent B): 15–36% B (21 min), 36–78% (14 min) followed by a cleaning cycle (78–100% B for 0.10 min, 2.5 min hold, 100–15% B for 0.1 min, 15% B for 1.5 min). The amino acids were quantified by using a calibration curve of the commercially available mix of standard amino acids (Fluka, Germany). For the polyamine analysis, samples were extracted as previously reported and analysed by HPLC-FLD as *o*-phathaldehyde (OPA)-ethanethiol (ET)-fluotenylmethyl chloroformate (FMOC) derivatives, according to Hanczko *et al*. (2005). A Chromolith Performance RP-18e column (4.6 mm i.d., Merck, Germany) was used at a flow rate of 1.5 ml min^−1^ at 28°C with the following gradient of 0.02 M citrate buffer of pH 5.5 (solvent A) and methanol : acetonitrile (65:35, v : v; solvent B): 0–100% B (10 min), 100% B (13 min), 50% B for 0.10 min, 50% for 1.5 min. The polyamines were quantified by using a calibration curve of spermine, spermidine and putrescine (Sigma Aldrich, Germany).
