# Supplementary material for: The MAP kinase MpkA controls cell wall integrity, oxidative stress response, gliotoxin production and iron adaptation in Aspergillus fumigatus
Source: Mol Microbiol. 2011 Aug 30;82(1):39–53. doi: 10.1111/j.1365-2958.2011.07778.x (PMC3229709; doi:10.1111/j.1365-2958.2011.07778.x)
Supplement: Supplementary file 1 [file mmi0082-0039-SD1.pdf]

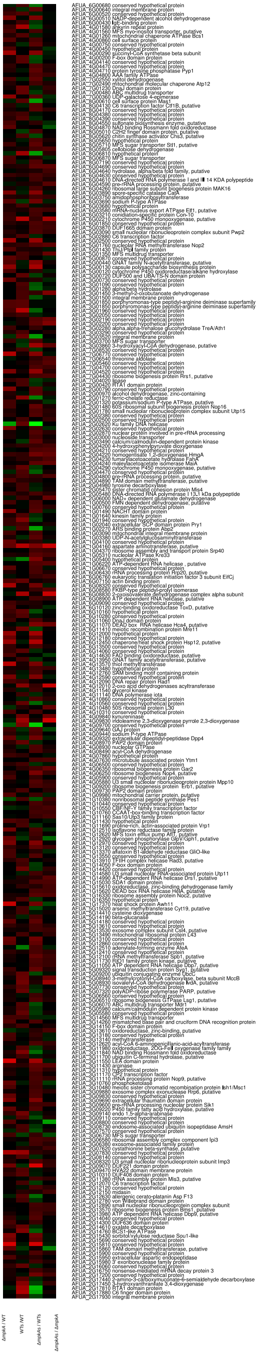
AFUA_6G00680 conserved hypothetical protein
AFUA_6G00640 integral membrane protein
AFUA_6G00520 conserved hypothetical protein
AFUA_6G00510 NADP-dependent alcohol dehydrogenase
AFUA_6G00430 IgE-binding protein
AFUA_4G01580 ankyrin repeat protein
AFUA_4G01560 MFS myo-inositol transporter, putative
AFUA_4G01260 mitochondrial chaperone ATPase Bcs1
AFUA_4G00860 cell surface protein
AFUA_4G00750 conserved hypothetical protein
AFUA_4G00450 hypothetical protein
AFUA_4G00290 succinyl-CoA synthetase beta subunit
AFUA_4G00200 F-box domain protein
AFUA_4G04140 conserved hypothetical protein
AFUA_4G04470 conserved hypothetical protein
AFUA_4G04710 protein tyrosine phosphatase Pyp1
AFUA_4G04800 AAA family ATPase
AFUA_7G02550 xylitol dehydrogenase
AFUA_7G02490 mitochondrial molecular chaperone Atp12
AFUA_7G01230 DnaJ domain protein
AFUA_7G00480 ABC multidrug transporter
AFUA_7G00360 UDP-galactose 4-epimerase
AFUA_8G00610 cell surface protein Mas1
AFUA_8G04130 C6 transcription factor Ctf1B, putative
AFUA_8G04170 conserved hypothetical protein
AFUA_8G04380 conserved hypothetical protein
AFUA_8G04390 conserved hypothetical protein
AFUA_8G04550 sulfonate biosynthesis enzyme, putative
AFUA_8G04870 NAD binding Rossmann fold oxidoreductase
AFUA_8G05010 C2H2 finger domain protein, putative
AFUA_8G05620 chitin synthase activator Chs3, putative
AFUA_8G05650 hypothetical protein
AFUA_8G05710 MFS sugar transporter Stl1, putative
AFUA_8G05805 cellobiose dehydrogenase
AFUA_8G06810 hypothetical protein
AFUA_8G06870 MFS sugar transporter
AFUA_8G07190 conserved hypothetical protein
AFUA_6G04690 conserved hypothetical protein
AFUA_6G04640 hydrolase, alpha/beta fold family, putative
AFUA_6G04630 conserved hypothetical protein
AFUA_6G04610 DNA-directed RNA polymerase I and III 14 KDA polypeptide
AFUA_6G04590 pre-rRNA processing protein, putative
AFUA_6G04280 ribosomal large subunit biogenesis protein MAK16
AFUA_6G03890 spore-specific catalase CatA
AFUA_6G03750 amidophosphoribosyltransferase
AFUA_6G03690 sodium P-type ATPase
AFUA_6G03680 hypothetical protein
AFUA_6G03580 mRNA-nucleus export ATPase Elf1, putative
AFUA_6G03210 conidiation-specific protein Con-10
AFUA_6G02210 cytochrome P450 monooxygenase, putative
AFUA_6G01850 conserved hypothetical protein
AFUA_5G03870 DUF1665 domain protein
AFUA_5G03090 small nucleolar ribonucleoprotein complex subunit Pwp2
AFUA_5G02880 C6 transcription factor
AFUA_5G02500 conserved hypothetical protein
AFUA_5G01760 nucleolar RNA methyltransferase Nop2
AFUA_5G01430 ThiJ/PfpI family protein
AFUA_5G01350 MFS multidrug transporter
AFUA_5G00870 conserved hypothetical protein
AFUA_5G00440 GNAT family N-acetyltransferase, putative
AFUA_3G00130 capsule polysaccharide biosynthesis protein
AFUA_3G00120 cytochrome P450 oxidoreductase/alkane hydroxylase
AFUA_3G00720 DUF500 and UBA/TS-N domain protein
AFUA_3G00730 conserved hypothetical protein
AFUA_3G01090 conserved hypothetical protein
AFUA_3G01280 alpha/beta hydrolase
AFUA_3G01450 3-methyl-2-oxobutanoate dehydrogenase
AFUA_3G01500 integral membrane protein
AFUA_3G01850 porphyromonas-type peptidyl-arginine deiminase superfamily
AFUA_3G01850 porphyromonas-type peptidyl-arginine deiminase superfamily
AFUA_3G01960 conserved hypothetical protein
AFUA_3G02050 conserved hypothetical protein
AFUA_3G02190 conserved hypothetical protein
AFUA_3G02200 conserved hypothetical protein
AFUA_3G02280 alpha,alpha-trehalose glucohydrolase TreA/Ath1
AFUA_3G03290 conserved hypothetical protein
AFUA_3G03520 integral membrane protein
AFUA_3G03700 MFS sugar transporter
AFUA_3G03860 3-hydroxyacyl-CoA dehydrogenase, putative
AFUA_7G08530 conserved hypothetical protein
AFUA_7G06770 conserved hypothetical protein
AFUA_7G06540 threonine aldolase
AFUA_7G05460 conserved hypothetical protein
AFUA_7G04700 conserved hypothetical protein
AFUA_7G04520 conserved hypothetical protein
AFUA_7G04430 ribosome biogenesis protein Rrs1, putative
AFUA_7G04020 lipase
AFUA_2G00420 RTA1 domain protein
AFUA_2G00790 conserved hypothetical protein
AFUA_2G00970 alcohol dehydrogenase, zinc-containing
AFUA_2G01270 ferric-chelate reductase
AFUA_2G01320 potassium/sodium P-type ATPase, putative
AFUA_2G01480 60S ribosomal subunit biogenesis protein Nop16
AFUA_2G01780 small nucleolar ribonucleoprotein complex subunit Utp15
AFUA_2G02380 conserved hypothetical protein
AFUA_2G02500 conserved hypothetical protein
AFUA_2G02620 Ku family DNA helicase
AFUA_2G02630 conserved hypothetical protein
AFUA_2G02870 nuclear protein involved in pre-rRNA processing
AFUA_2G03000 nucleoside transporter
AFUA_2G03490 calcium/calmodulin-dependent protein kinase
AFUA_2G04200 4-hydroxyphenylpyruvate dioxygenase
AFUA_2G04210 conserved hypothetical protein
AFUA_2G04220 homogentisate 1,2-dioxygenase HmgA
AFUA_2G04230 fumarylacetoacetate hydrolase FahA
AFUA_2G04240 maleylacetoacetate isomerase MaiA
AFUA_2G04290 cytochrome P450 monooxygenase, putative
AFUA_2G04470 conserved hypothetical protein
AFUA_2G04800 pre-rRNA processing protein, putative
AFUA_2G04890 TAM domain methyltransferase, putative
AFUA_2G04980 tyrosine decarboxylase
AFUA_2G05470 sister chromatid cohesion protein Mis4
AFUA_2G05480 DNA-directed RNA polymerase I 13.1 kDa polypeptide
AFUA_2G06000 NAD+ dependent glutamate dehydrogenase
AFUA_1G00500 FMN dependent dehydrogenase, putative
AFUA_1G00760 conserved hypothetical protein
AFUA_1G01490 NACHT domain protein
AFUA_1G01640 kinesin family protein
AFUA_1G01940 conserved hypothetical protein
AFUA_1G02040 extracellular SCP domain protein Pry1
AFUA_1G02270 ARS binding protein Abp2
AFUA_1G03090 mitochondrial integral membrane protein
AFUA_1G03380 UDP-N-acetylglucosaminyltransferase
AFUA_1G04100 conserved hypothetical protein
AFUA_1G04160 aspartate aminotransferase, putative
AFUA_1G04370 ribosome assembly and transport protein Srp40
AFUA_1G05310 nucleolar ATPase Kre33
AFUA_1G05400 hypothetical protein
AFUA_1G06220 ATP-dependent RNA helicase , putative
AFUA_1G06670 conserved hypothetical protein
AFUA_6G06430 rRNA processing protein Rrp20, putative
AFUA_6G06760 eukaryotic translation initiation factor 3 subunit EifCj
AFUA_6G07150 actin binding protein
AFUA_6G08320 conserved hypothetical protein
AFUA_6G08580 FKBP-type peptidyl-prolyl isomerase
AFUA_6G08830 2-oxoisovalerate dehydrogenase complex alpha subunit
AFUA_6G08900 ATP dependent RNA helicase, putative
AFUA_6G09090 conserved hypothetical protein
AFUA_6G10120 zinc-binding oxidoreductase ToxD, putative
AFUA_6G10160 hypothetical protein
AFUA_6G10280 conserved hypothetical protein
AFUA_6G11060 DnaJ domain protein
AFUA_6G11070 DEAD box RNA helicase Hca4, putative
AFUA_6G11410 meiotic recombination protein Mre11
AFUA_6G12000 hypothetical protein
AFUA_6G12180 conserved hypothetical protein
AFUA_6G12450 chaperone/heat shock protein Hsp12, putative
AFUA_6G13500 conserved hypothetical protein
AFUA_6G14060 conserved hypothetical protein
AFUA_4G14630 FAD binding oxidoreductase, putative
AFUA_4G13950 GNAT family acetyltransferase, putative
AFUA_4G13570 thiol methyltransferase
AFUA_4G13480 hypothetical protein
AFUA_4G12760 SAM binding motif containing protein
AFUA_4G12590 conserved hypothetical protein
AFUA_4G12090 DNA repair protein Rad1
AFUA_4G12010 2-oxo acid dehydrogenases acyltransferase
AFUA_4G11540 glycerol kinase
AFUA_4G11140 DNA polymerase iota
AFUA_4G10860 conserved hypothetical protein
AFUA_4G10560 conserved hypothetical protein
AFUA_4G10480 50S ribosomal protein L30
AFUA_4G10310 conserved hypothetical protein
AFUA_4G09840 kynureninase
AFUA_4G09830 indoleamine 2,3-dioxygenase pyrrole 2,3-dioxygenase
AFUA_4G09700 conserved hypothetical protein
AFUA_4G09640 GAJ protein
AFUA_4G09440 sodium P-type ATPase
AFUA_4G09320 extracellular dipeptidyl-peptidase Dpp4
AFUA_4G08970 PAP2 domain protein
AFUA_4G08930 nucleolar GTPase
AFUA_4G08490 acyl-CoA dehydrogenase
AFUA_4G07860 hypothetical protein
AFUA_4G07630 microtubule associated protein Ytm1
AFUA_4G06500 conserved hypothetical protein
AFUA_4G06350 ribosomal biogenesis protein Gar2
AFUA_4G06250 ribosome biogenesis protein Nop4, putative
AFUA_4G05900 conserved hypothetical protein
AFUA_4G05880 U3 small nucleolar ribonucleoprotein protein Mpp10
AFUA_1G09200 ribosome biogenesis protein Erb1, putative
AFUA_1G09730 PAP2 domain protein
AFUA_1G09980 mitochondrial carrier protein, putative
AFUA_1G10380 nonribosomal peptide synthase Pes1
AFUA_1G10440 conserved hypothetical protein
AFUA_1G10550 CBF/NF-Y family transcription factor
AFUA_1G10760 CCAAT-box-binding transcription factor
AFUA_1G11160 Sas10/Utp3 family protein
AFUA_1G11430 hypothetical protein
AFUA_1G11690 proline-rich, actin-associated protein Vrp1
AFUA_1G12510 isoflavone reductase family protein
AFUA_1G12620 MFS toxin efflux pump AflT, putative
AFUA_1G12920 glycogen phosphorylase GlpV/Gph1, putative
AFUA_1G12970 conserved hypothetical protein
AFUA_1G13120 conserved hypothetical protein
AFUA_1G13270 aflatoxin B1-aldehyde reductase GliO-like
AFUA_1G13550 conserved hypothetical protein
AFUA_1G13910 TFIIH complex helicase Rad3, putative
AFUA_1G14050 F-box domain protein
AFUA_1G14420 conserved hypothetical protein
AFUA_1G14580 U3 small nucleolar RNA-associated protein Utp11
AFUA_1G14990 ATP-dependent RNA helicase Drs1, putative
AFUA_1G15030 SDA1 domain protein
AFUA_1G15610 oxidoreductase, zinc-binding dehydrogenase family
AFUA_1G15620 DEAD box RNA helicase HelA, putative
AFUA_1G16330 ribosome assembly protein Noc2, putative
AFUA_1G16350 hypothetical protein
AFUA_1G17370 heat shock protein Awh11
AFUA_5G15020 arsenic methyltransferase Cyt19, putative
AFUA_5G14410 cysteine dioxygenase
AFUA_5G14190 beta-glucanase
AFUA_5G14180 conserved hypothetical protein
AFUA_5G13610 conserved hypothetical protein
AFUA_5G13530 exosome complex subunit Csl4, putative
AFUA_5G13490 mitochondrial ribosomal protein L43
AFUA_5G13100 conserved hypothetical protein
AFUA_5G12860 conserved hypothetical protein
AFUA_5G12510 adenylate-forming enzyme AfeA
AFUA_5G12410 conserved hypothetical protein
AFUA_5G12170 rRNA methyltransferase Spb1, putative
AFUA_5G11730 RIO1 family protein kinase, putative
AFUA_5G11050 ATP dependent RNA helicase Dbp7, putative
AFUA_5G09320 signal transduction protein Syg1, putative
AFUA_5G09200 ubiquitin conjugating enzyme UbcC
AFUA_5G08940 3-methylcrotonyl-CoA carboxylase, beta subunit MccB
AFUA_5G08930 isovaleryl-CoA dehydrogenase IvdA, putative
AFUA_5G07730 conserved hypothetical protein
AFUA_5G07320 polyADP-ribose polymerase PARP, putative
AFUA_5G06560 conserved hypothetical protein
AFUA_5G06510 ribosome biogenesis GTPase Lsg1, putative
AFUA_5G06070 ABC multidrug transporter Mdr1
AFUA_5G05980 calcium/calmodulin dependent protein kinase
AFUA_5G05580 conserved hypothetical protein
AFUA_5G05450 conserved hypothetical protein
AFUA_5G04560 MFS multidrug transporter
AFUA_3G14260 mismatched base pair and cruciform DNA recognition protein
AFUA_3G14150 F-box domain protein
AFUA_3G13610 oxidoreductase, zinc-binding dehydrogenase family
AFUA_3G13180 conserved hypothetical protein
AFUA_3G13140 methyltransferase
AFUA_3G12620 acyl-CoA 6-aminopenicillanic-acid-acyltransferase
AFUA_3G11980 polyketide synthase, 2OG-FeII oxygenase family
AFUA_3G11840 NAD binding Rossmann fold oxidoreductase
AFUA_3G11700 ubiquitin C-terminal hydrolase, putative
AFUA_3G11550 LEA domain protein
AFUA_3G11430 arginase
AFUA_3G11310 hypothetical protein
AFUA_3G11170 CP2 transcription factor
AFUA_3G11110 rRNA processing protein Nop9, putative
AFUA_3G10760 phosphoketolase
AFUA_3G10480 meiotic sister chromatid recombination protein Ish1/Msc1
AFUA_3G09880 exosome complex exonuclease Rrp6, putative
AFUA_3G09830 conserved hypothetical protein
AFUA_3G09690 extracellular thaumatin domain protein
AFUA_3G09600 pre-rRNA processing nucleolar protein Sik1
AFUA_3G09220 P450 family fatty acid hydroxylase, putative
AFUA_3G09140 endo 1,5-alpha-arabinase
AFUA_3G09110 conserved hypothetical protein
AFUA_3G08800 conserved hypothetical protein
AFUA_3G08770 conserved hypothetical protein
AFUA_3G08730 endosome-associated ubiquitin isopeptidase AmsH
AFUA_3G07570 conserved hypothetical protein
AFUA_3G06730 MFS sugar transporter
AFUA_3G06580 ribosomal assembly complex component Ipi3
AFUA_3G06380 exosome-associated family protein
AFUA_2G07620 cystathionine beta-synthase, putative
AFUA_2G07830 conserved hypothetical protein
AFUA_2G08140 conserved hypothetical protein
AFUA_2G08320 U3 small nucleolar ribonucleoprotein subunit Imp3
AFUA_2G09070 DUF221 domain protein
AFUA_2G09470 HVA22 domain membrane protein
AFUA_2G10310 DUF408 domain protein
AFUA_2G11380 rRNA assembly protein Mis3, putative
AFUA_2G12070 C6 transcription factor
AFUA_2G12120 conserved hypothetical protein
AFUA_2G12150 midasin
AFUA_2G12630 allergenic cerato-platanin Asp F13
AFUA_2G12780 von Willebrand domain protein
AFUA_2G12890 small nucleolar ribonucleoprotein complex subunit
AFUA_2G13570 ribosome biogenesis protein Bms1, putative
AFUA_2G13980 ATP dependent RNA helicase Dbp9, putative
AFUA_2G14040 conserved hypothetical protein
AFUA_2G14300 DUF636 domain protein
AFUA_2G14610 oxalate decarboxylase
AFUA_2G14760 BCS1-like ATPase
AFUA_2G15430 sorbitol/xylulose reductase Sou1-like
AFUA_2G15690 conserved hypothetical protein
AFUA_2G15810 conserved hypothetical protein
AFUA_2G15860 TAM domain methyltransferase, putative
AFUA_2G15900 conserved hypothetical protein
AFUA_2G15950 extracellular aspartic endopeptidase
AFUA_2G15980 3' exoribonuclease family protein
AFUA_2G16060 conserved hypothetical protein
AFUA_2G16750 nonsense-mediated mRNA decay protein 3
AFUA_2G17200 conserved hypothetical protein
AFUA_2G17440 2-amino-3-carboxymuconate-6-semialdehyde decarboxylase
AFUA_2G17450 3-hydroxyanthranilate 3,4-dioxygenase
AFUA_2G17810 RTA1 domain protein
AFUA_2G17880 C6 finger domain protein
AFUA_2G17930 integral membrane protein
ΔmpkA / WT
WTs /WT
ΔmpkAs / WTs
ΔmpkAs / ΔmpkA

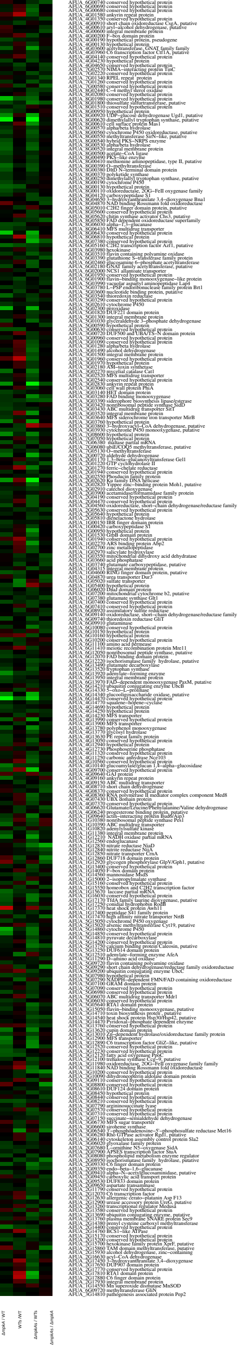
AFUA_6G00740 conserved hypothetical protein
AFUA_6G00690 conserved hypothetical protein
AFUA_6G00680 conserved hypothetical protein
AFUA_4G01580 ankyrin repeat protein
AFUA_4G01350 conserved hypothetical protein
AFUA_4G00910 short chain oxidoreductase CsgA, putative
AFUA_4G00610 aryl−alcohol dehydrogenase, putative
AFUA_4G00600 integral membrane protein
AFUA_4G00200 F−box domain protein
AFUA_4G00190 hypothetical protein, pseudogene
AFUA_4G00130 hypothetical protein
AFUA_4G03600 acetyltransferase, GNAT family family
AFUA_4G03960 C6 transcription factor Ctf1A, putative
AFUA_4G04140 conserved hypothetical protein
AFUA_4G04230 hypothetical protein
AFUA_4G04650 conserved hypothetical protein
AFUA_7G02570 NIMA−interacting protein TinC
AFUA_7G02220 conserved hypothetical protein
AFUA_7G01340 RPEL repeat protein
AFUA_7G01260 conserved hypothetical protein
AFUA_7G00580 conserved hypothetical protein
AFUA_8G02440 C−4 methyl sterol oxidase
AFUA_8G02080 conserved hypothetical protein
AFUA_8G01980 conserved hypothetical protein
AFUA_8G01800 thiosulfate sulfurtransferase, putative
AFUA_8G01510 conserved hypothetical protein
AFUA_8G00950 hypothetical protein
AFUA_8G00920 UDP−glucose dehydrogenase Ugd1, putative
AFUA_8G00620 dimethylallyl tryptophan synthase, putative
AFUA_8G00610 cell surface protein Mas1
AFUA_8G00570 alpha/beta hydrolase
AFUA_8G00560 cytochrome P450 oxidoreductase, putative
AFUA_8G00550 methyltransferase SirN−like, putative
AFUA_8G00540 hybrid PKS−NRPS enzyme
AFUA_8G00530 alpha/beta hydrolase
AFUA_8G00520 integral membrane protein
AFUA_8G00500 acetate−CoA ligase
AFUA_8G00490 PKS−like enzyme
AFUA_8G00410 methionine aminopeptidase, type II, putative
AFUA_8G00390 O−methyltransferase
AFUA_8G00380 DltD N−terminal domain protein
AFUA_8G00370 polyketide synthase
AFUA_8G00250 dimethylallyl tryptophan synthase, putative
AFUA_8G00190 cytochrome P450
AFUA_8G00130 hypothetical protein
AFUA_8G00110 oxidoreductase, 2OG−FeII oxygenase family
AFUA_8G04120 carboxypeptidase S1
AFUA_8G04650 3−hydroxyanthranilate 3,4−dioxygenase Bna1
AFUA_8G04870 NAD binding Rossmann fold oxidoreductase
AFUA_8G05010 C2H2 finger domain protein, putative
AFUA_8G05600 conserved hypothetical protein
AFUA_8G05620 chitin synthase activator Chs3, putative
AFUA_8G05850 FAD dependent oxidoreductase superfamily
AFUA_8G06030 alpha−1,3−glucanase
AFUA_8G06410 MFS multidrug transporter
AFUA_8G06430 conserved hypothetical protein
AFUA_8G06810 hypothetical protein
AFUA_8G07380 conserved hypothetical protein
AFUA_6G05160 C2H2 transcription factor Azf1, putative
AFUA_6G03980 hexokinase
AFUA_6G03510 flavin containing polyamine oxidase
AFUA_6G03390 glutathione S−transferase family protein
AFUA_6G02460 glucosamine 6−phosphate acetyltransferase
AFUA_6G02180 GNAT family acetyltransferase, putative
AFUA_6G02000 NCS1 allantoate transporter
AFUA_6G01950 conserved hypothetical protein
AFUA_6G01900 flavin−binding monooxygenase−like protein
AFUA_5G03990 vacuolar aspartyl aminopeptidase Lap4
AFUA_5G03780 L−PSP endoribonuclease family protein Brt1
AFUA_5G03600 nucleotide binding protein, putative
AFUA_5G03540 thioredoxin reductase
AFUA_5G03290 conserved hypothetical protein
AFUA_5G02610 cytochrome P450
AFUA_5G02300 peroxidase
AFUA_5G01820 DUF221 domain protein
AFUA_5G01300 integral membrane protein
AFUA_5G01030 glyceraldehyde 3−phosphate dehydrogenase
AFUA_5G00590 hypothetical protein
AFUA_3G00630 conserved hypothetical protein
AFUA_3G00720 DUF500 and UBA/TS−N domain protein
AFUA_3G00960 conserved hypothetical protein
AFUA_3G01090 conserved hypothetical protein
AFUA_3G01280 alpha/beta hydrolase
AFUA_3G01490 alcohol dehydrogenase
AFUA_3G01500 integral membrane protein
AFUA_3G01960 conserved hypothetical protein
AFUA_3G01970 hypothetical protein
AFUA_3G02180 AM−toxin synthetase
AFUA_3G02270 mycelial catalase Cat1
AFUA_3G02520 MFS multidrug transporter
AFUA_3G02540 conserved hypothetical protein
AFUA_3G02830 ankyrin repeat protein
AFUA_3G03060 cell wall protein PhiA
AFUA_3G03140 HET domain protein
AFUA_3G03280 FAD binding monooxygenase
AFUA_3G03390 siderophore biosynthesis lipase/esterase
AFUA_3G03420 nonribosomal peptide synthase SidD
AFUA_3G03430 ABC multidrug transporter SitT
AFUA_3G03520 integral membrane protein
AFUA_3G03640 MFS siderochrome iron transporter MirB
AFUA_3G03760 hypothetical protein
AFUA_3G03860 3−hydroxyacyl−CoA dehydrogenase, putative
AFUA_3G03930 cytochrome P450 monooxygenase, putative
AFUA_7G08600 hypothetical protein
AFUA_7G07050 hypothetical protein
AFUA_7G06380 maltase partial mRNA
AFUA_7G06080 ubiE/COQ5 methyltransferase, putative
AFUA_7G05130 O−methyltransferase
AFUA_2G00720 aldehyde dehydrogenase
AFUA_2G01170 1,3−beta−glucanosyltransferase Gel1
AFUA_2G01220 GTP cyclohydrolase II
AFUA_2G01270 ferric−chelate reductase
AFUA_2G01940 conserved hypothetical protein
AFUA_2G02550 Phosducin family protein
AFUA_2G02620 Ku family DNA helicase
AFUA_2G02820 Yippee zinc−binding protein Moh1, putative
AFUA_2G02910 catechol dioxygenase
AFUA_2G03900 acetamidase/formamidase family protein
AFUA_2G04190 conserved hypothetical protein
AFUA_2G04470 conserved hypothetical protein
AFUA_2G04560 oxidoreductase, short−chain dehydrogenase/reductase family
AFUA_2G05630 conserved hypothetical protein
AFUA_2G05640 hypothetical protein
AFUA_2G05810 dienelactone hydrolase
AFUA_1G00150 IBR finger domain protein
AFUA_1G00420 carboxypeptidase S1
AFUA_1G00950 hypothetical protein
AFUA_1G01530 GrpB domain protein
AFUA_1G01940 conserved hypothetical protein
AFUA_1G02270 ARS binding protein Abp2
AFUA_1G02700 zinc metallopeptidase
AFUA_1G02970 salicylate hydroxylase
AFUA_1G03550 mitochondrial dihydroxy acid dehydratase
AFUA_1G03660 acid phosphatase
AFUA_1G03740 glutamate carboxypeptidase, putative
AFUA_1G04315 integral membrane protein
AFUA_1G04600 RING finger domain protein, putative
AFUA_1G04870 urea transporter Dur3
AFUA_1G05020 sulfate transporter
AFUA_1G05400 hypothetical protein
AFUA_1G06020 DnaJ domain protein
AFUA_1G07200 mitochondrial cytochrome b2, putative
AFUA_1G07380 glutamate synthase Glt1
AFUA_1G07400 conserved hypothetical protein
AFUA_6G07410 conserved hypothetical protein
AFUA_6G08920 assimilatory sulfite reductase
AFUA_6G09140 oxidoreductase, short−chain dehydrogenase/reductase family
AFUA_6G09740 thioredoxin reductase GliT
AFUA_6G09910 glutaminase
AFUA_6G10080 conserved hypothetical protein
AFUA_6G10150 hypothetical protein
AFUA_6G10160 hypothetical protein
AFUA_6G10200 conserved hypothetical protein
AFUA_6G11100 amino acid permease
AFUA_6G11410 meiotic recombination protein Mre11
AFUA_6G12050 nonribosomal peptide synthase, putative
AFUA_6G12070 FAD binding domain protein
AFUA_6G12220 isochorismatase family hydrolase, putative
AFUA_6G13490 glutamate decarboxylase
AFUA_6G13520 tryptophan synthase
AFUA_6G13920 adenylate−forming enzyme
AFUA_6G13950 integral membrane protein
AFUA_6G13970 FAD−dependent monooxygenase PaxM, putative
AFUA_6G14210 ubiquitin conjugating enzyme UbcB
AFUA_6G14330 5−oxo−L−prolinase
AFUA_6G14340 glucooligosaccharide oxidase, putative
AFUA_6G14470 conserved hypothetical protein
AFUA_4G14770 squalene−hopene−cyclase
AFUA_4G14690 hypothetical protein
AFUA_4G14250 hypothetical protein
AFUA_4G14230 MFS transporter
AFUA_4G13990 conserved hypothetical protein
AFUA_4G13900 MFS transporter
AFUA_4G13780 polyphenol monooxygenase
AFUA_4G13770 glycosyl hydrolase
AFUA_4G13630 PE repeat family protein
AFUA_4G13050 conserved hypothetical protein
AFUA_4G12940 hypothetical protein
AFUA_4G12730 Phosphoserine phosphatase
AFUA_4G11320 conserved hypothetical protein
AFUA_4G11250 carbonic anhydrase Nce103
AFUA_4G10560 conserved hypothetical protein
AFUA_4G10140 glucoamylase/glucan 1,4−alpha−glucosidase
AFUA_4G09700 conserved hypothetical protein
AFUA_4G09640 GAJ protein
AFUA_4G09160 ankyrin repeat protein
AFUA_4G09150 ABC multidrug transporter
AFUA_4G08710 short chain dehydrogenase
AFUA_4G08370 conserved hypothetical protein
AFUA_4G08360 RNA polymerase II mediator complex component Med8
AFUA_4G07430 UBX domain protein
AFUA_4G07370 conserved hypothetical protein
AFUA_4G06620 Glutamate/Leucine/Phenylalanine/Valine dehydrogenase
AFUA_4G06240 progesterone binding protein, putative
AFUA_1G09640 actin−interacting protein Bud6/Aip3
AFUA_1G10380 nonribosomal peptide synthase Pes1
AFUA_1G10390 ABC multidrug transporter
AFUA_1G10820 adenylylsulfate kinase
AFUA_1G11380 integral membrane protein
AFUA_1G12210 NADH oxidase partial mRNA
AFUA_1G12560 endoglucanase
AFUA_1G12830 nitrate reductase NiaD
AFUA_1G12840 nitrite reductase NiiA
AFUA_1G12850 nitrate transporter CrnA
AFUA_1G12860 DUF718 domain protein
AFUA_1G12920 glycogen phosphorylase GlpV/Gph1, putative
AFUA_1G13400 conserved hypothetical protein
AFUA_1G14050 F−box domain protein
AFUA_1G14560 mannosidase MsdS
AFUA_1G15000 2−isopropylmalate synthase
AFUA_1G15160 conserved hypothetical protein
AFUA_1G15550 homeobox and C2H2 transcription factor
AFUA_1G15670 laccase partial mRNA
AFUA_1G16030 conserved hypothetical protein
AFUA_1G17170 TfdA family taurine dioxygenase, putative
AFUA_1G17250 conidial hydrophobin RodB
AFUA_1G17370 heat shock protein Awh11
AFUA_1G17400 peptidase S41 family protein
AFUA_1G17470 high affinity nitrate transporter NrtB
AFUA_5G15050 cytochrome P450 oxygenase
AFUA_5G15020 arsenic methyltransferase Cyt19, putative
AFUA_5G14860 cytochrome P450
AFUA_5G14850 conserved hypothetical protein
AFUA_5G14810 pyruvate decarboxylase
AFUA_5G14200 conserved hypothetical protein
AFUA_5G13750 calcium binding protein Caleosin, putative
AFUA_5G13250 DUF614 domain protein
AFUA_5G12510 adenylate−forming enzyme AfeA
AFUA_5G11290 D−amino acid oxidase
AFUA_5G09720 flavin containing polyamine oxidase
AFUA_5G09290 short chain dehydrogenase/reductase family oxidoreductase
AFUA_5G09200 ubiquitin conjugating enzyme UbcC
AFUA_5G07980 hypothetical protein
AFUA_5G07290 NADPH−dependent FMN/FAD containing oxidoreductase
AFUA_5G07100 GRAM domain protein
AFUA_5G07090 conserved hypothetical protein
AFUA_5G06980 conserved hypothetical protein
AFUA_5G06070 ABC multidrug transporter Mdr1
AFUA_5G06030 conserved hypothetical protein
AFUA_5G05640 RTA1 domain protein
AFUA_3G15050 flavin−binding monooxygenase, putative
AFUA_3G14710 toxin biosynthesis proten , putative
AFUA_3G14540 heat shock protein Hsp30/Hsp42, putative
AFUA_3G14470 Pyridoxal−phosphate dependent enzyme
AFUA_3G13760 conserved hypothetical protein
AFUA_3G13620 cupin domain protein
AFUA_3G13010 Zn−dependent hydrolase/oxidoreductase family protein
AFUA_3G12900 MFS transporter
AFUA_3G12890 C6 transcription factor GliZ−like, putative
AFUA_3G12520 conserved hypothetical protein
AFUA_3G12230 conserved hypothetical protein
AFUA_3G12120 fatty acid oxygenase PpoC
AFUA_3G12100 trehalose synthase Ccg−9, putative
AFUA_3G11980 oxidoreductase, 2OG−FeII oxygenase family family
AFUA_3G11840 NAD binding Rossmann fold oxidoreductase
AFUA_3G10200 conserved hypothetical protein
AFUA_3G10090 dihydroneopterin aldolase domain protein
AFUA_3G09110 conserved hypothetical protein
AFUA_3G08800 conserved hypothetical protein
AFUA_3G08610 DUF124 domain protein
AFUA_3G08450 hypothetical protein
AFUA_3G08440 conserved hypothetical protein
AFUA_3G08210 conserved hypothetical protein
AFUA_3G07790 argininosuccinate lyase
AFUA_3G07570 conserved hypothetical protein
AFUA_3G07310 conserved hypothetical protein
AFUA_3G07150 succinate−semialdehyde dehydrogenase
AFUA_3G06730 MFS sugar transporter
AFUA_3G06600 siroheme synthase
AFUA_3G06540 3'−phosphoadenosine−5'−phosphosulfate reductase Met16
AFUA_3G06280 Rho GTPase activator Rgd1, putative
AFUA_3G06140 cytoskeleton assembly control protein Sla2
AFUA_3G06020 glyoxalase family protein
AFUA_2G07680 L−ornithine N5−oxygenase SidA
AFUA_2G07900 APSES transcription factor StuA
AFUA_2G08080 phospholipid metabolism enzyme regulator
AFUA_2G08950 isochorismatase family hydrolase, putative
AFUA_2G09330 C6 finger domain protein
AFUA_2G09350 endo−beta−1,6−glucanase
AFUA_2G09410 alpha−N−acetylglucosaminidase, putative
AFUA_2G09450 carboxylic acid transport protein
AFUA_2G09530 DUF833 domain protein
AFUA_2G09650 aspartate transaminase
AFUA_2G11790 conserved hypothetical protein
AFUA_2G12070 C6 transcription factor
AFUA_2G12630 allergenic cerato−platanin Asp F13
AFUA_2G12900 urease accessory protein UreG, putative
AFUA_2G13260 transcriptional regulator Medusa
AFUA_2G13580 conserved hypothetical protein
AFUA_2G13690 ubiquitin conjugating enzyme, putative
AFUA_2G13760 plasma membrane SNARE protein Sec9
AFUA_2G14380 prenyl cysteine carboxyl methyltransferase
AFUA_2G14400 conserved hypothetical protein
AFUA_2G14760 BCS1−like ATPase
AFUA_2G15170 conserved hypothetical protein
AFUA_2G15200 conserved hypothetical protein
AFUA_2G15700 hexokinase family protein XprF, putative
AFUA_2G15860 TAM domain methyltransferase, putative
AFUA_2G15930 alcohol dehydrogenase, zinc−containing
AFUA_2G16630 acyl−CoA dehydrogenase
AFUA_2G17450 3−hydroxyanthranilate 3,4−dioxygenase
AFUA_2G17650 DUF907 domain protein
AFUA_2G17770 conserved hypothetical protein
AFUA_2G17810 RTA1 domain protein
AFUA_2G17880 C6 finger domain protein
AFUA_2G17930 integral membrane protein
AFUA_1G14550 Mn superoxide dismutase MnSOD
AFUA_6G09720 methyltransferase GliN
AFUA_3G14810 pathogenesis associated protein Pep2
ΔmpkA / WT
WTs /WT
ΔmpkAs / WTs
ΔmpkAs / ΔmpkA

Supplementary Fig. 3

Acc num	name	A/W	Ws/W	As/Ws	As/A
	Nitrate-assimilation cluster				
AFUA_1G12830	nitrate reductase NiaD	1,13	-1,87	-3,97	-7,99
AFUA_1G12840	nitrite reductase NiiA	1,05	-1,36	-2,00	-2,75
AFUA_1G12850	nitrate transporter CrnA	1,05	-1,36	-1,46	-2,01
	pyomelanin cluster	A/W	Ws/W	As/Ws	As/A
AFUA_2G04190	conserved hypothetical protein	-1,86	1,10	-2,87	-1,39
AFUA_2G04200	4-hydroxyphenylpyruvate dioxygenase	1,43	1,65	1,82	2,06
AFUA_2G04210	conserved hypothetical protein	-1,19	-1,07	2,25	2,50
AFUA_2G04220	homogentisate 1,2-dioxygenase HmgA	1,85	1,65	3,38	2,93
AFUA_2G04230	umarylacetoacetate hydrolase FahA	1,26	1,68	1,77	2,28
AFUA_2G04240	maleylacetoacetate isomerase MaiA	1,10	1,20	2,75	2,89
	gli-like cluster	A/W	Ws/W	As/Ws	As/A
AFUA_3G12890	C6 transcription factor GliZ-like	-3,72	1,12	-4,73	-1,13
AFUA_3G12900	MFS transporter	-4,30	-1,10	-3,85	1,03
AFUA_3G12910	O-methyltransferase GliM-like, putative	-1,32	-1,03	-1,31	-1,02
AFUA_3G12920	nonribosomal peptide synthase GliP-like, putative	-1,25	-1,36	1,24	1,14
AFUA_3G12930	dimethylallyl tryptophan synthase SirD-like, putative	-1,66	1,05	-1,79	-1,02
AFUA_3G12940	C6 transcription factor, putative	1,01	-1,06	-1,06	-1,13
AFUA_3G12950	FAD binding domain protein	-1,36	-1,11	-1,14	1,07
AFUA_3G12960	cytochrome P450 monooxigenase GliC-like, putative	-1,61	-1,23	-1,26	1,06
	Tryptophan metabolism	A/W	Ws/W	As/Ws	As/A
AFUA_4G09820	histone acetyltransferase subunit Yaf9	1,01	1,13	-1,22	-1,09
AFUA_4G09830	indoleamine 2,3-dioxygenase pyrrole 2,3-dioxygenase	0,73	5,29	1,33	9,18
AFUA_4G09840	kynureninase	-1,20	3,20	-1,13	3,15
	Leu degradation cluster	A/W	Ws/W	As/Ws	As/A
AFUA_5G08900	D-arabinitol dehydrogenase ArbD, putative	-1,23	-1,18	-1,36	-1,28
AFUA_5G08910	3-methylcrotonyl-CoA carboxylase subunit alpha (MccA), putative	-1,27	-1,35	1,06	1,00
AFUA_5G08920	conserved hypothetical protein	1,65	1,32	1,53	1,22
AFUA_5G08930	isovaleryl-CoA dehydrogenase IvdA, putative	-1,15	3,41	1,43	5,13
AFUA_5G08940	3-methylcrotonyl-CoA carboxylase, beta subunit (MccB), putative	1,20	3,00	1,28	3,00
	Pseurostatin A	A/W	Ws/W	As/Ws	As/A
AFUA_8G00370	polyketide synthase	-2,02	-1,17	-1,76	-1,01
AFUA_8G00380	DltD N-terminal domain protein	-2,97	-1,08	-2,88	-1,04
AFUA_8G00390	O-methyltransferase	-3,68	-1,62	-2,79	-1,19
AFUA_8G00400	conserved hypothetical protein	-1,20	1,05	-1,27	-1,01
AFUA_8G00410	methionine aminopeptidase, type II, putative	-2,06	-1,12	-2,09	-1,12
AFUA_8G00420	C6 finger transcription factor, putative	-1,80	-1,74	-1,81	-1,70
AFUA_8G00430	conserved hypothetical protein	-1,47	-1,07	-1,31	1,05
AFUA_8G00440	steroid monooxygenase	-1,85	1,07	-1,92	-0,97
AFUA_8G00460	methionine aminopeptidase, type I, putative	-1,33	-1,15	-1,38	-1,17
AFUA_8G00470	conserved hypothetical protein	1,03	1,29	-1,43	-1,14
AFUA_8G00480	phytanoyl-CoA dioxygenase family protein	1,25	1,11	-1,09	-1,23
AFUA_8G00490	PKS-like enzyme	-2,04	1,06	-2,32	-1,06
AFUA_8G00500	acetate-CoA ligase	-2,56	-1,24	-1,86	1,12
AFUA_8G00510	cytochrome P450 oxidoreductase OrdA-like	-1,51	-1,07	-1,44	-1,02
AFUA_8G00520	integral membrane protein	-3,25	-1,26	-3,39	-1,28
AFUA_8G00530	alpha/beta hydrolase	-3,01	-1,10	-3,07	-1,11
AFUA_8G00540	hybrid PKS-NRPS enzyme	-2,48	-1,12	-2,19	1,02
AFUA_8G00550	methyltransferase SirN-like, putative	-1,69	1,10	-2,55	-1,36
AFUA_8G00560	cytochrome P450 oxidoreductase, putative	-2,39	1,10	-3,02	-1,14
AFUA_8G00570	alpha/beta hydrolase	-1,94	1,23	-2,25	1,06
AFUA_8G00580	glutathione S-transferase	-1,68	-1,19	-1,39	1,02
AFUA_8G00590	fatty acid desaturase	-1,32	1,31	-1,71	1,01
AFUA_8G00600	NAD dependent epimerase/dehydratase family protein	1,11	1,65	-1,23	1,19
AFUA_8G00610	cell surface protein Mas1	-1,51	2,57	-5,43	-1,42
AFUA_8G00620	dimethylallyl tryptophan synthase, putative	-17,43	-1,17	-19,53	-1,26

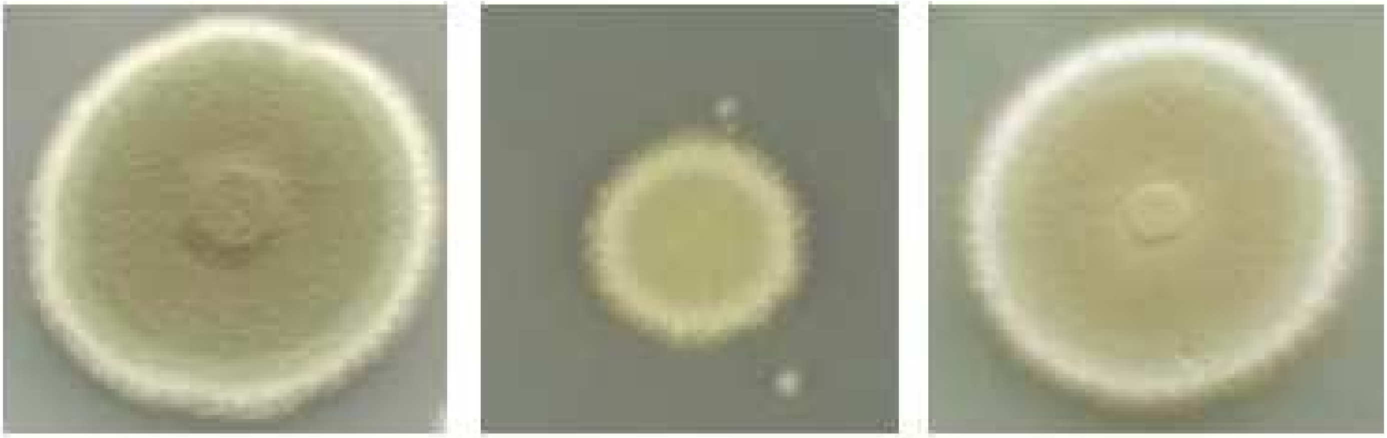

Wild type | Δ*mpkA* | Δ*mpkA*/*mpkA egfp*

Supplementary Fig. 4

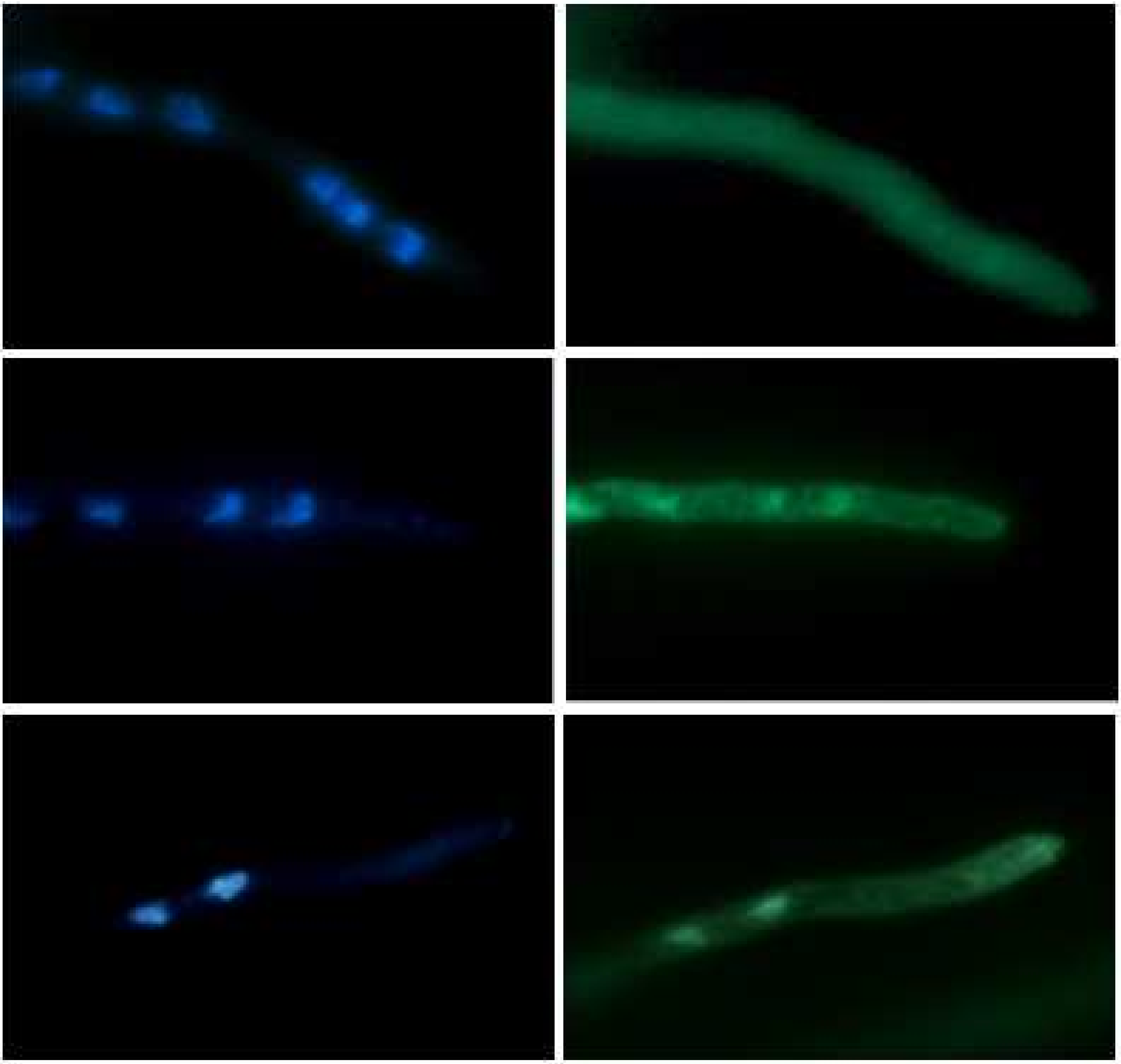

Supplementary Figure 5

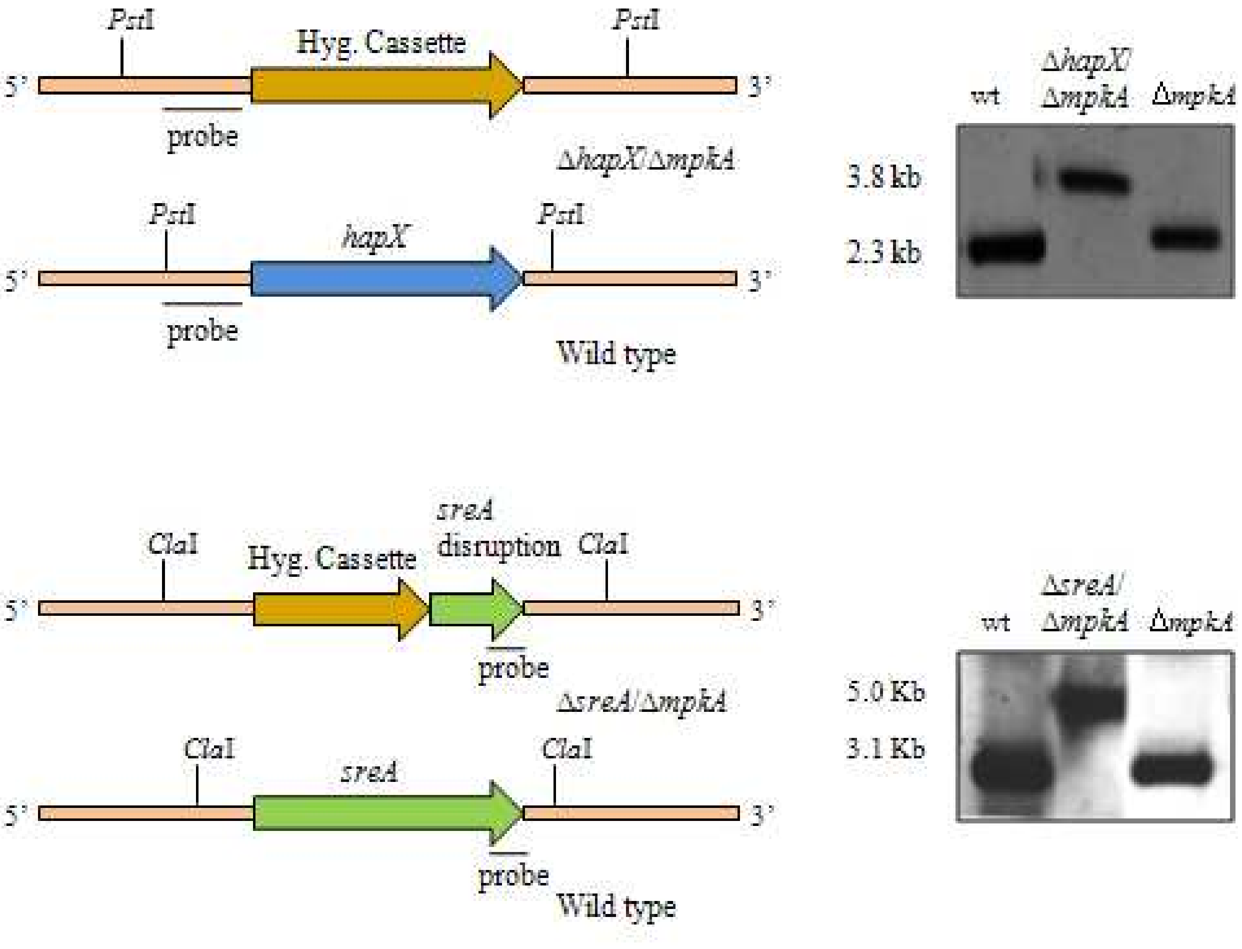

Supplementary Figure 6

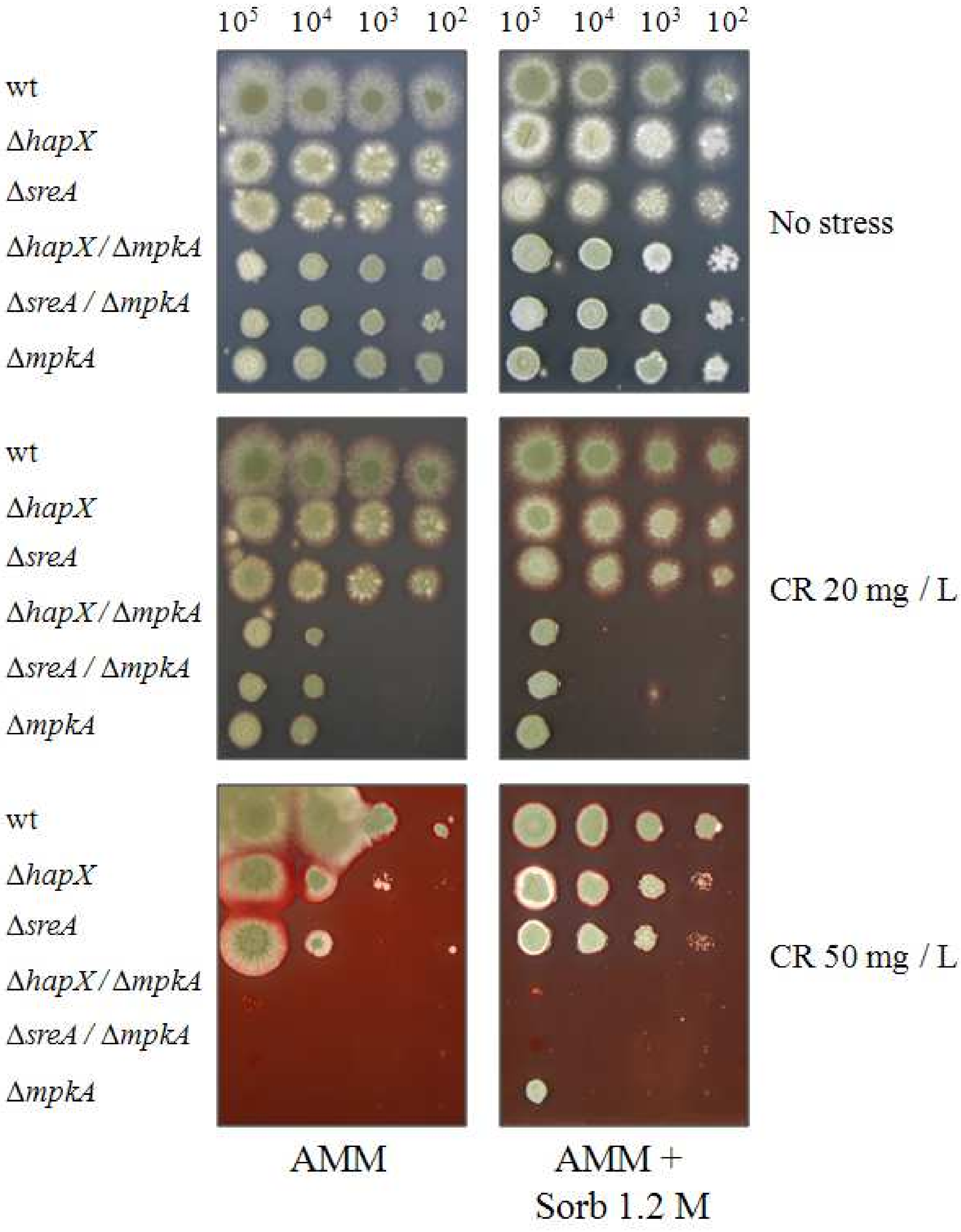

Supplementary Fig. 7

Suppementary Table 1

Oligonucletides used in this study

Oligonucleotides	Sequence 5´ – 3´
Hph_for	GTGGCACTATTGATCATCCG
Hph_rev	GCCAGTGTGCTGGAATTCGC
HapX_ for_1	GGGTATCACCACCGTATCCAGGC
HapX_ rev_1	CGGAGCCAGGGCAGGAGTGGATG
HapX_rev(hph)	CGGATGATCAATAGTGCCACGCGATCTGGATGGACATTAGTGG
HapX_for(hph)	GCGAATTCCAGCACACTGGCGATGATACCCAAACGGACTCG
SreA_for_2	AACCCGCATGTCTAAGCC
SreA_rev_2	CACGCAGCACTGAATCAC
Cat1_for_1	GTCGGCGATGGCTTCTTCAC
Cat1_rev_1	GCCACATACACGCCGTCCC
Gel1_for_1	CCGCAAGTACCGTGAGATTCC
Gel1_rev_1	GCGCCGAAGACGGTGGACAG
SidA_for_1	GTGGTCAGAGTGCTGCGGAG
SidA_rev_1	CGCCACCACGAACAGCC
HapX_for_2	GCTCCAGTTCCCTTGCCTCTCC
HapX_rev_2	GGTGGAGGAGAATGGTAGGG
SreA_ for_1	GTTGCTACCGACCCACCACG
SreA_rev_1	GTCTAGGCGGGATTCGTCGTGC
SidC_ for_1	GGCATTCAGCCGACTTGCACACC
SidC_ rev_1	CCATCCCACGCACCTCCTTCTC
HemA_for	CACGTCTCCCACCGCTCTGC
HemA_rev	CGGTCACCCGCTCTTCCATC
Orn amt_for_1	CGCAGCCCACAACTATCACCCTCTC
Orn amt_rev_1	CGACAATGCCTGCTTCGCCCTG
Orn dec_for_2	GCCGTAGACGCCATCGTTCAGG
Orn dec_rev_2	GCCGCCGAGGTGGATGTGGACC
SpdA_for_2	GTCCGAGATCACTCACCCCAC
SpdA_rev_2	CACATCCAGAGCCTTGCGTGC
Arg_for_1	GGGTCCAAGGCGTCAACGTCG
Arg_rev_1	CTCACACTAGGCGGCGACCAC
AmcA_for_1	GCTGCCTGTCCTGCCACCTAAC
AmcA_rev_1	GCCTCGGTACAATGCCCTCAGC
MpkA_for_1	CGCGGGATCCATGTCTGATCTACAGGGTCG
MpkA_Sma1_rev	CCCGGGTTGGACATCCATCCCCCGCTG
MnSOD_for_1	CACAGCAATACACGCTCCCACC
MnSOD_rev_1	CGCTATGTACCGATTCTCCG
RodB_for_1	CGTCCACCCTACCTTCGCCAGCG
RodB_rev_1	GAGTCGAGAGCAACGCAGGC
MsdS_for_1	GCTGTCTGGATACGACCTGCTC
MsdS_rev_1	CATTCCACACCCAGTCACGG

Supplementary Figure 1

Heatmap showing the pattern of expression of all upregulated genes of *A. fumigatus* in four different comparisons. Column 1, Δ*mpkA*/wt; Column 2, wt + glucanex/wt; Column 3, Δ*mpkA* + glucanex/wt + glucanex; Column 4, Δ*mpkA* + glucanex/Δ*mpkA*.

Supplementary Figure 2

Heatmap showing the pattern of expression of all downregulated genes in *A. fumigatus* under four different comparisons. Column 1, Δ*mpkA*/wt; Column 2, wt + glucanex/wt; Column 3, Δ*mpkA* + glucanex/wt + glucanex; Column 4, Δ*mpkA* + glucanex/Δ*mpkA*.

Supplementary Figure 3

Secondary metabolite gene clusters and genes required for metabolism which were found to be differentially regulated by microarray analysis. Fold change ≤ -1.5 is represented by grey box whereas fold change ≥ 1.5 is represented by black box.

Supplementary Figure 4

Colony phenotype of wild type, Δ*mpkA* and Δ*mpkA*/*mpkA egfp*. 10^4 conidia were spotted on AMM agar plates and incubated at 37 °C for 4 days.

Supplementary Figure 5

(A) Localization of MpkA under standard conditions, **(B)** cell wall stress (glucanex) and **(C)** ROS stress induced by hydrogen peroxide.

Supplementary Figure 6

Genome organization and Southern blot analyses of Δ*hap*X/Δ*mpkA* and Δ*sreA*/Δ*mpkA* mutants.

Supplementary Figure 7

Sensitivity of the strains Δ*hapX*, Δ*sreA*, Δ*hapX* / Δ*mpkA* , Δ*sreA* / Δ*mpkA* and Δ*mpkA* to cell wall-disturbing compounds. The indicated number of conidia was spotted on AMM agar plates, or AMM agar plates supplemented with congo red (CR). In order to attempt to rescue the mutant phenotypes by adding osmoregulators, agar plates were additionally supplemented with 1,2 M sorbitol (right panels). All the agar plates were incubated at 37 °C for 2 to 3 days.

Supplementary Table 1

Oligonucleotides used in this study.
